# Overexpressed Pseudogene *HLA-DPB2* Promotes Tumor Immune Infiltrates by Regulating *HLA-DPB1* and Indicates a Better Prognosis in Breast Cancer

**DOI:** 10.3389/fonc.2020.01245

**Published:** 2020-08-07

**Authors:** Lijuan Lyu, Jia Yao, Meng Wang, Yi Zheng, Peng Xu, Shuqian Wang, Dai Zhang, Yujiao Deng, Ying Wu, Si Yang, Jun Lyu, Feng Guan, Zhijun Dai

**Affiliations:** ^1^Department of Breast Surgery, The First Affiliated Hospital, College of Medicine, Zhejiang University, Hangzhou, China; ^2^Department of Oncology, The Second Affiliated Hospital of Xi'an Jiaotong University, Xi'an, China; ^3^Department of Clinical Research, The First Affiliated Hospital of Jinan University, Guangzhou, China; ^4^Provincial Key Laboratory of Biotechnology, Joint International Research Laboratory of Glycobiology and Medicinal Chemistry, College of Life Science, Northwest University, Xi'an, China

**Keywords:** pseudogene, *HLA-DPB2*, *HLA-DPB1*, breast cancer, prognosis, immune infiltration

## Abstract

Immune checkpoint inhibitors (ICIs) have been successfully used for treating melanoma and non-small cell lung cancer. However, many patients with breast cancer (BC) show low response to ICIs due to the paucity of infiltrating immune cells. Pseudogenes, as a particular kind of long-chain noncoding RNA, play vital roles in tumorigenesis, but their potential roles in tumor immunology remain unclear. In this study that used data from online databases, the novel pseudogene *HLA-DPB2* and its parental gene *HLA-DPB1* were overexpressed and correlated with better prognosis in BC. Mechanistically, our results revealed that *HLA-DPB2* might serve as an endogenous RNA to increase *HLA-DPB1* expression by competitively binding with *has-miR-370-3p*. Functionally, gene ontology (GO) and Kyoto Encyclopedia of Genes and Genomes enrichment analysis indicated that the *HLA-DPB2/HLA-DPB1* axis was strongly relevant to immune-related biological functions. Further analysis demonstrated that high expression levels of the *HLA-DPB2* and *HLA-DPB1* were significantly associated with high immune infiltration abundance of CD8+ T cells, CD4+ T cells, Tfh, Th1, and NK cells and with high expression of majority biomarkers of monocytes, NK cell, T cell, CD8+ T cell, and Th1 in BC and its subtype, indicating that *HLA-DPB2* can increase the abundance of tumor-infiltrating lymphocytes in the BC microenvironment. Also, the *HLA-DPB2* and *HLA-DPB1* expression levels positively correlated with the expression levels of programmed cell death protein 1, programmed cell death ligand 1, and cytotoxic T-lymphocyte-associated antigen-4. Our findings suggest that pseudogene *HLA-DPB2* can upregulate *HLA-DPB1* through sponging has-miR-370-3p, thus exerting its antitumor effect by recruiting tumor-infiltrating immune cells into the breast tumor microenvironment, and that targeting the *HLA-DPB2/HLA-DPB1* axis with ICIs may optimize the current immunotherapy for BC.

## Introduction

Breast cancer (BC) is the leading cancer that affects women, and its incidence rate is clearly increasing in recent years (1). Despite huge advances in the early detection and early diagnosis and the combination of multiple treatments, the mortality rate of BC is still increasing significantly worldwide, and BC remains a global burden (2–4). Recently, tumor immunity and immunotherapy have attracted extensive attention in the treatment of multiple solid cancers and have been successful in the clinical field as a treatment for melanoma and non-small cell lung cancer (5). Immunotherapy is gradually becoming the future development direction of cancer treatment and is called the fourth major treatment technology for BC after surgery, radiotherapy, and chemotherapy (6).

Tumor immunotherapy is to overcome the mechanism of tumor immune escape, thereby reawakening immune cells to clear cancer cells, including immune system modulators, tumor antigen vaccines, adoptive cellular therapy, and immune checkpoint inhibitors (ICIs) (7). A recent research study has revealed the better therapeutic effect of programmed cell death protein 1/programmed cell death ligand 1 (PD-1/PD-L1) antagonists in combination with nab-paclitaxel in metastatic triple-negative BC (TNBC) (8). Unfortunately, most patients with BC, such as hormone-positive BCs, showed low response rates to the current immunotherapies such as PD-1 and PD-L1 inhibitors due to the paucity of infiltrating immune cells, which is called the “cold” immunological nature of BC (9). Consequently, to observe the dramatic response to immunotherapy in BC as has been observed in melanoma, finding ways to turn immunologically “cold” tumors to “hot” tumors by, such as increasing the abundances of tumor-infiltrating lymphocytes (TILs) in the microenvironment is imperative (10). TILs are predictive markers of the tumor-immune microenvironment and the response to ICIs therapy (11). Emerging studies have shown that the greater number of TILs, the stronger response to ICIs therapy (12, 13). The future of immunotherapy in BC lies in the combination of ICIs with strategies that activate the immune system to pursue maximal antitumor efficacy (14).

Pseudogenes, traditionally regarded as “junk genes” or “genomic fossils” owing to their lack of protein-coding ability, are a class of long noncoding RNAs (lncRNAs) that control the expressions of their homologous protein-coding genes (parent genes) or irrelevant genes by binding with various DNAs, RNAs, or proteins (15). Recently, mounting evidence has suggested that pseudogenes are often dysregulated in diverse human tumors, which can lead to the onset and progression of cancers (16, 17). For instance, the pseudogene *PTENP1* acts as a competing endogenous RNA (ceRNA) to control *PTEN* expression, which mediates malignant behaviors of multiple cancers, including BC (18–22). Besides, high *PTTG3P* expression level promoted tumor cell proliferation, migration, and invasion and indicated bad prognosis in BC (23), cervical cancer (24), gastric cancer (25), and esophageal squamous cell carcinoma (26). Specially, Yang et al. (27) found that the pseudogene *RP11-424C20.2* acted as a ceRNA to increase its parental gene *UHRF1* expression, which obviously associated with immune infiltration in hepatocellular carcinoma and thymoma. However, evidence on the function of pseudogenes in tumor immunity remains sparse.

In the present study, we first identified a novel BC prognosis-associated pseudogene *HLA-DPB2*, of which the expression, prognosis, role, and corresponding regulatory mechanisms of *HLA-DPB2* in BC have not been illuminated. *HLA-DPB1*, the parental gene of *HLA-DPB2*, is part of human leukocyte antigen (HLA) complex and generally expressed in antigen-presenting cells (28). Previous research studies reported that its parental gene, *HLA-DPB1*, can promote immunity and is essential for immunotherapy in leukemia (29, 30). Nevertheless, the expression and potential roles of *HLA-DPB1* in solid tumors have not been reported, and the regulatory relationship between *HLA-DPB2* and *HLA-DPB1* in BC also has not been elucidated. Therefore, we conducted this study to analyze the expression and prognostic values of the pseudogene *HLA-DPB2* and its parental gene *HLA-DPB1* in BC by mining a series of databases. Then, we examined several potential mechanisms of *HLA-DPB2* in BC, including the regulatory mechanism between *HLA-DPB2* and its parental gene *HLA-DPB1*, performed functional enrichment analysis, and constructed a protein–protein interaction (PPI) network of the top 100 correlated genes. Finally, we investigated the correlation of *HLA-DPB2/HLA-DPB1* expression with immune infiltration in BC.

## Materials and Methods

### Identification of Differentially Expressed Pseudogenes Related to Prognosis

The high-throughput sequencing data of pseudogenes in BC were directly obtained from dreamBase (http://rna.sysu.edu.cn/dreamBase/) (31). The thresholds for differential expression was set at |fold change| ≥ 2.0. Subsequently, the UALCAN (http://ualcan.path.uab.edu/analysis.html) (32) database was used to analyze the prognostic significance of these differentially expressed pseudogenes in BC. Finally, the screened pseudogenes related to prognosis were used in a follow-up analysis.

### Gene Expression Analysis Using a Series of Databases

We first determined the *HLA-DPB2* and *HLA-DPB1* expression profiles using ONCOMINE (https://www.oncomine.org/resource/main) (33). We conducted the ONCOMINE analysis of tumor samples of 20 cancer types and normal samples and also performed a meta-analysis of datasets in BC. The cutoff was defined as: *P*-values of <0.0001, a fold change of >2.0, and the gene rank >10%. Then, the *HLA-DPB2* and *HLA-DPB1* expression levels were validated using data from the TIMER (https://cistrome.shinyapps.io/timer/) (34) and starBase (http://starbase.sysu.edu.cn/) (35) databases. Also, we investigated the association of HLA-DPB2 and HLA-DPB1 messenger RNA (mRNA) levels with the clinicopathological features of BC by using bc-GenExMiner v4.4 (http://bcgenex.centregauducheau.fr/) (36). Finally, the correlation of pseudogene *HLA-DPB2* and its parental gene *HLA-DPB1* was analyzed by using data from the starBase (35), UALCAN (32), bc-GenExMiner (36), and TIMER (34) databases. *P*-values of <0.05 were considered statistically significant.

### Prognostic Analysis Using Data From the OncoLnc and Kaplan–Meier Plotter Databases

OncoLnc (http://www.oncolnc.org/) (37) was applied to evaluate the relationship between the gene expression of *HLA-DPB2* or *HLA-DPB1* and the overall survival (OS) of patients with BC. Kaplan–Meier Plotter (http://kmplot.com/analysis/) (38) was introduced to assess the associations of *HLA-DPB2* or *HLA-DPB1* expression with OS and relapse-free survival (RFS) in BC by using pan-cancer RNA-seq data and those of *HLA-DPB1* expression with OS, RFS, distant metastases-free survival (DMFS), and post-progression survival (PPS) by using microarray data of BC. We then downloaded clinical data and RNA-seq data of *HLA-DPB2* and *HLA-DPB1* of BC patients from The Cancer Genome Atlas (39) database by the Genomic Data Commons website (https://portal.gdc.cancer.gov/) and performed Cox survival regression analysis to evaluate the dependent prognostic value of mRNA expression of *HLA-DPB2* and *HLA-DPB1* in terms of OS in BC patients. Log-rank *P*-values of <0.05 were considered statistically significant.

### Pseudogene *HLA-DPB2* Subcellular Localization Prediction

The sequence of *HLA-DPB2* was extracted from the National Center for Biotechnology Information, and its subcellular localization was explored by its sequence using IncLocator (http://www.csbio.sjtu.edu.cn/bioinf/lncLocator/) (40), which can predict five subcellular localizations of lncRNAs, namely the cytoplasm, nucleus, cytosol, ribosome, and exosome.

### Prediction of Candidate MicroRNAs of *HLA-DPB2* and *HLA-DPB1*

First, we applied miRanda (http://www.microrna.org/) (41) to determine potential microRNAs (miRNAs) binding to the pseudogene *HLA-DPB2* 3′ untranslated region. Subsequently, potential binding miRNAs of *HLA-DPB1* 3′ untranslated region were predicted using TargetScanHuman7.2 (http://www.targetscan.org/) (42) and miRWalk (43). Then, we analyzed the potential miRNAs using Venn's diagram. The expression level of the candidate miRNA in BC was detected using dbDEMC2 (https://www.picb.ac.cn/dbDEMC/) (44). *P*-value < 0.05 was considered statistically significant.

### Protein–Protein Interaction Network and Gene Ontology and Kyoto Encyclopedia of Genes and Genomes Enrichment Analysis

The top 100 correlated genes with the *HLA-DPB2* and *HLA-DPB1* were downloaded from UALCAN (32). The PPI network for these correlated genes was built using STRING v11.0 (https://string-db.org/) (45) and visualized by Cytoscape v3.8. Metascape (http://metascape.org/) (46) was introduced to conduct gene ontology (GO) and Kyoto Encyclopedia of Genes and Genomes pathway enrichment analysis of the top 100 genes from UALCAN that correlated with the *HLA-DPB2* and *HLA-DPB1* in BC. Only terms with *p*-values of <0.01, minimum count of 3, and enrichment factor of >1.5 were considered significant.

### Correlation Analysis Between *HLA-DPB2/HLA-DPB1* Expression and Immune Infiltration

The Spearman correlation of *HLA-DPB2* or *HLA-DPB1* expression with the immune infiltration levels of B cells, CD4+ T cells, CD8+ T cells, neutrophils, macrophages, and DCs in BC and its subtype was visualized using the “Gene” module in TIMER (34). The Spearman correlation of *HLA-DPB2/HLA-DPB1* expression with the immune marker sets of tumor-infiltrating immune cells and expressions of PD-1, PD-L1, and cytotoxic T-lymphocyte-associated antigen-4 (CTLA-4) was visualized using the “Correlation” module. The gene markers of a tumor-infiltrating immune cell are referenced in prior studies (47, 48). The correlation was adjusted by tumor purity. Moreover, we used TISIDB (http://cis.hku.hk/TISIDB/) (49) to verify the correlation of *HLA-DPB1* expression with the abundance of 28 TILs and expressions of PD-1, PD-L1, and CTLA-4 and to analyze the distribution of *HLA-DPB1* expression across immune subtypes of BC.

## Results

### Screening for Differentially Expressed Pseudogenes Related With Prognosis in Breast Cancer

Firstly, we screened differentially expressed pseudogenes in BC using dreamBase. Based on the cutoff criteria, 264 upregulated and 368 downregulated pseudogenes were finally confirmed in BC (Supplementary Table 1). Subsequently, we determined the expression profiles and prognostic significance of these dysregulated pseudogenes in BC using UALCAN. Except that most gene symbols were not identified in UALCAN, 21 of 22 upregulated pseudogenes and 29 of 34 downregulated pseudogenes were lined with the analytical results from the dreamBase, as listed in Supplementary Table 2. Among these pseudogenes, only *HLA-DPB2* expression was associated with the patient prognosis in BC (Supplementary Figure 1, *P* < 0.05). Therefore, *HLA-DPB2* was selected as a candidate pseudogene for further analysis.

### Overexpressions of *HLA-DPB2* and *HLA-DPB1* Predict Better Survival in Breast Cancer

**Figure 1:**
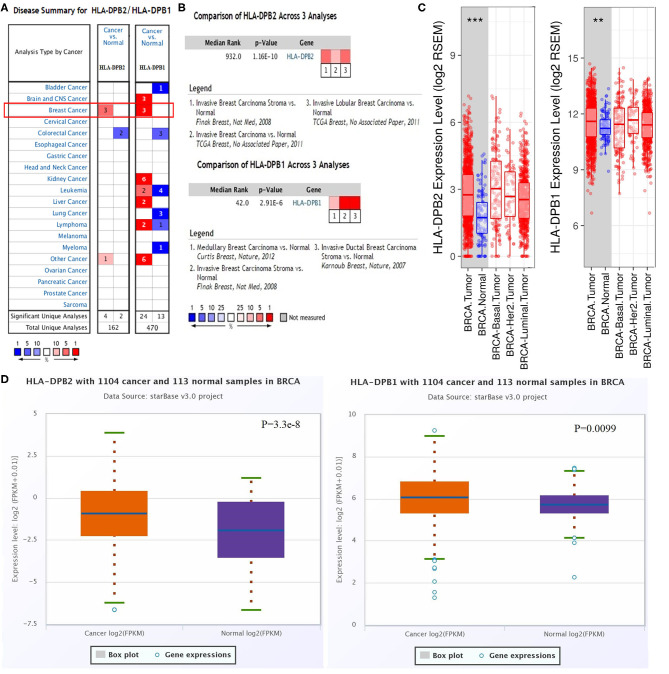
Pseudogene *HLA-DPB2* and its parental gene *HLA-DPB1* are upregulated in BC tissues. **(A)** Expression of *HLA-DPB2* and *HLA-DPB1* in different types of cancers by ONCOMINE analysis of cancer vs. normal samples; **(B)** meta-analysis of *HLA-DPB2* and *HLA-DPB1* expression in BC using ONCOMINE database; **(C,D)** the messenger RNA expression level of *HLA-DPB2* and *HLA-DPB1* in BC samples compared with normal tissues using TIMER and starBase databases, respectively. BC, breast cancer; **P < 0.01, ***P < 0.001.

**Figure 2:**
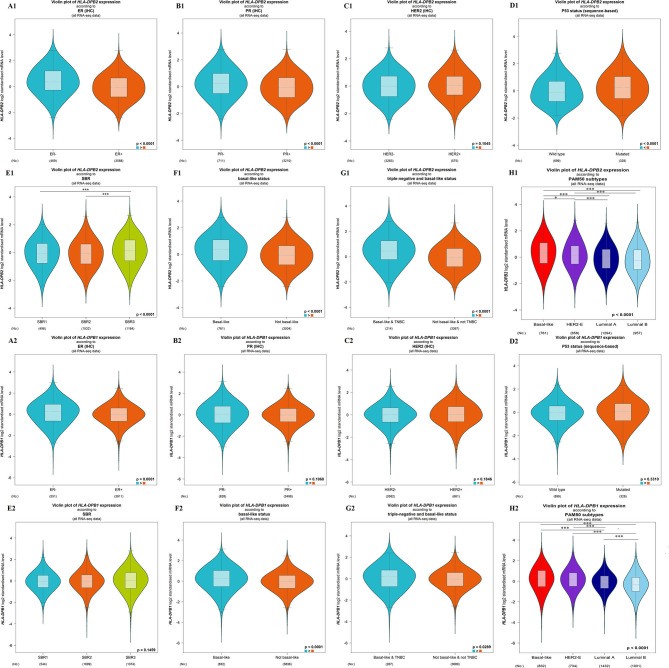
Association of mRNA expression of *HLA-DPB2* and *HLA-DPB1* with clinicopathological characteristics in BC patients using bc-GenExMiner database. **(A1,A2)** Association of *HLA-DPB2* and *HLA-DPB1* expression with estrogen receptor status; **(B1,B2)** Association of *HLA-DPB2* and *HLA-DPB1* expression with progesterone receptor status; **(C1,C2)** Association of *HLA-DPB2* and *HLA-DPB1* expression with human epidermal growth factor receptor-2 (HER2) status; **(D1,D2)** Association of *HLA-DPB2* and *HLA-DPB1* expression with P53 status; **(E1,E2)** Association of *HLA-DPB2* and *HLA-DPB1* expression with SBR grade; **(F1,F2)** Association of *HLA-DPB2* and *HLA-DPB1* expression with basal-like status; **(G1,G2)** Association of *HLA-DPB2* and *HLA-DPB1* expression with triple negative and basal-like status; **(H1,H2)** Association of *HLA-DPB2* and *HLA-DPB1* expression with PAM50 subtypes. Difference of mRNA expression was compared by Welch's tests and Dunnett-Tukey-Kramer's test. BC, breast cancer; *P < 0.05, ***P < 0.001.

The microarray data from the ONCOMINE database were applied to analyze the expression pattern of the pseudogene *HLA-DPB2* and its parental gene *HLA-DPB1* (Figure 1A). We further applied the ONCOMINE meta-analysis to evaluate the comprehensive expression level of *HLA-DPB2* and *HLA-DPB1* across three datasets (Figure 1B). The details were shown in Supplementary Figure 2; compared with normal breast tissues, the expression level of the pseudogene *HLA-DPB2* significantly increased in invasive breast carcinoma (BRCA), invasive lobular breast carcinoma, and BRCA stroma (Supplementary Figures 2A–C, *P* < 0.0001), whereas mRNA expression of *HLA-DPB1* obviously enhanced in BRCA, invasive ductal breast carcinoma, and medullary breast carcinoma (Supplementary Figures 2D–F, *P* < 0.0001). Next, the mRNA expression level of *HLA-DPB2* and *HLA-DPB1* was further measured using the TIMER (Figure 1C) and starBase (Figure 1D) databases, whose resources were based on The Cancer Genome Atlas database, consistent with the ONCOMINE analysis. Compared with normal tissues, *HLA-DPB2* and *HLA-DPB1* mRNA expressions were upregulated in the BC group (*P* < 0.01). We further explored the relationship between the pseudogene *HLA-DPB2* and *HLA-DPB1* expression and some clinicopathological features of BC using bc-GenExMiner. We discovered that high mRNA level of *HLA-DPB2* was related to estrogen receptor-negative (ER-negative) (Figure 2A1, *P* < 0.0001), progesterone receptor-negative (Figure 2B1, *P* < 0.0001), P53-mutated (Figure 2D1, *P* < 0.0001), high Scarff–Bloom–Richardson (SBR) grade (Figure 2E1, *P* < 0.0001), and basal-like BC (Figures 2F1–H1, *P* < 0.0001), and that increased mRNA expression of *HLA-DPB1* was associated with ER-negative and basal-like BC (Figure 2A2, *P* < 0.0001 and Figures 2F2–H2, *P* < 0.05). However, there was no significant correlation of *HLA-DPB2* with human epidermal growth factor receptor-2 (HER2) status (Figure 2C1, *P* > 0.05) and *HLA-DPB1* with PR status, HER2 status, P53 status, and SBR grade (Figures 2B2–E2, *P* > 0.05).

**Figure 3:**
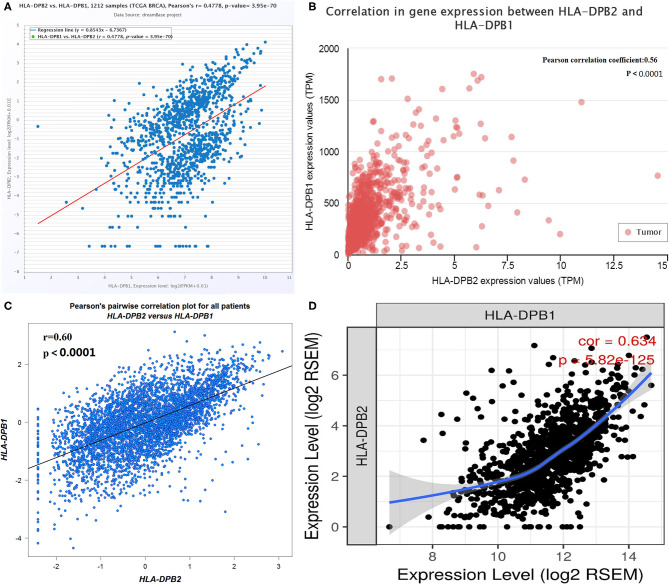
Correlation of *HLA-DPB2* with its parental gene *HLA-DPB1* in BC. **(A–D)** Expression association between *HLA-DPB2* and *HLA-DPB1* in BC analyzed using dreamBase, UALCAN, bc-GenExMiner, and TIMER database. BC, breast cancer.

Pseudogenes have been demonstrated to control their parental genes in several ways (50, 51). *HLA-DPB1* is the parental gene of *HLA-DPB2*. The correlation of pseudogene *HLA-DPB2* with *HLA-DPB1* was first analyzed using data from several databases. As shown in Figure 3A, the dreamBase correlation analysis suggested a strongly positive relationship between *HLA-DPB2* and *HLA-DPB1* in BC (Figure 3A, Pearson's *r* = 0.4778, *P* < 0.001). Similar results were acquired using analytical data from the UALCAN (Figure 3B, Pearson's *r* = 0.56, *P* < 0.001), bc-GenExMiner (Figure 3C, Pearson's *r* = 0.60, *P* < 0.001), and TIMER databases (Figure 3D, Spearman's *r* = 0.634, *P* < 0.001).

**Figure 4:**
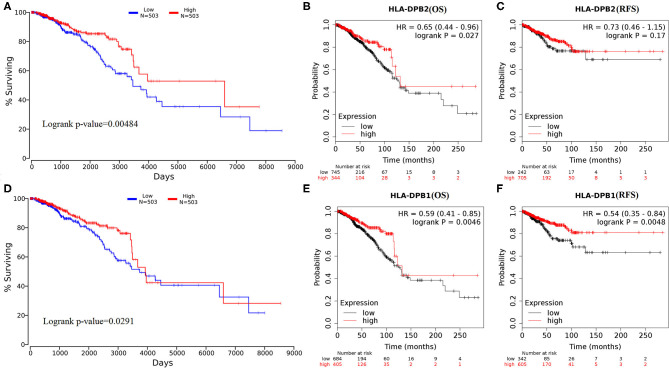
Overexpression of *HLA-DPB2* and *HLA-DPB1* is significantly associated with better prognosis in BC patients. **(A,D)** High expression of *HLA-DPB2* or *HLA-DPB1* indicates a better prognosis of BC by using OncoLnc database; **(B,E)** high expression of *HLA-DPB2* or *HLA-DPB1* indicates better OS in BC patients by Kaplan–Meier Plotter database; **(C)** high expression of *HLA-DPB2* is not related with RFS of BC patients by Kaplan–Meier Plotter database; **(F)** high expression of *HLA-DPB1* is related with RFS of BC patients by Kaplan–Meier Plotter database. BC, breast cancer; OS, overall survival; RFS, relapse-free survival.

Subsequently, the effect of *HLA-DPB2* and *HLA-DPB1* expression on patient survival of BC was evaluated using OncoLnc and Kaplan–Meier Plotter databases. The results suggested that high *HLA-DPB2* and *HLA-DPB1* expression levels were associated with better OS (*HLA-DPB2*, Figures 4A,B, *P* < 0.05; *HLA-DPB1*, Figures 4D,E and Supplementary Figures 3A1,A2, *P* < 0.05) in patients with BC. Moreover, high *HLA-DPB1* expression also indicated longer RFS (Figure 4F and Supplementary Figures 3B1,B2, *P* < 0.01) and DMFS (Supplementary Figures 3C1,C2, *P* < 0.05). However, high *HLA-DPB2* expression was not associated with RFS (Figure 4C, *P* > 0.05) of patients with BC. We then assess the independent prognostic value of mRNA expression of *HLA-DPB2* and *HLA-DPB1* in terms of OS in BC patients. In univariate analysis, we found that high mRNA expressions of *HLA-DPB2* [hazard ratio (HR) = 0.74, 95% CI: 0.60–0.92, and *P* = 0.006] and *HLA-DPB1* (HR = 0.995, 95% CI: 0.9918–0.9988, and *P* = 0.009) were related to longer OS of BC patients (Supplementary Table 3). Multivariate analysis also showed that increased mRNA expression expressions of *HLA-DPB2* (HR = 0.66, 95% CI: 0.51–0.86, and *P* = 0.002) and *HLA-DPB1* (HR = 0.996, 95% CI: 0.9928–0.9995, and *P* = 0.025) were independently associated with better OS of BC patients (Supplementary Table 3 and Supplementary Figure 4). Interestingly, the combination of *HLA-DPB2* with *HLA-DPB1* expressions was not associated with OS of BC patients (Supplementary Figure 7). These results suggested that mRNA expressions of *HLA-DPB2* and *HLA-DPB1* were independent prognostic factors for OS of BC patients and that *HLA-DPB2* expression in BC may have an anticancer effect in BC by regulating the expression of its parental gene, *HLA-DPB1*.

### *HLA-DPB2* Acts as a Sponge of *has-miR-370-3p* to Control *HLA-DPB1* Expression

**Figure 5:**
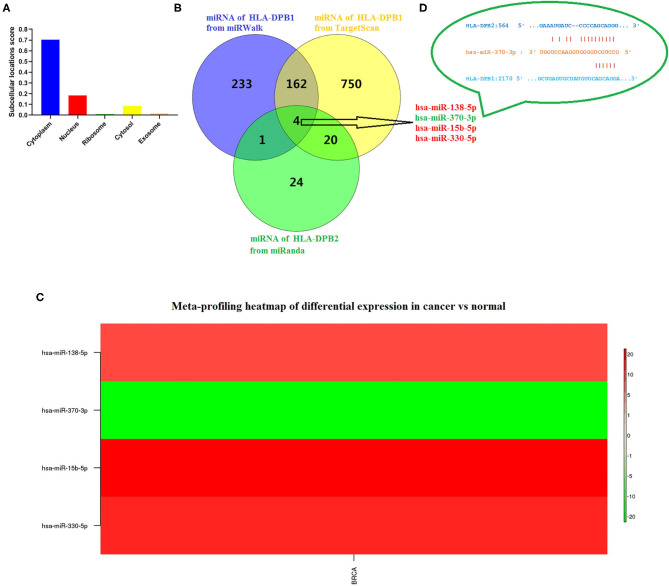
*Has-miR-370-3p* is identified as candidate miRNA for *HLA-DPB2* and *HLA-DPB1* in BC. **(A)** Prediction of subcellular localization of *HLA-DPB2* using lncLocator; **(B)** bioinformatics analysis of four candidate miRNAs for *HLA-DPB2* and *HLA-DPB1*; **(C)** meta-profiling of differential expression of four candidate miRNAs in BC tissues and normal tissues determined by dbDEMC 2; **(D)** base pairing between *has-miR-370-3p* and the putative target site in the *HLA-DPB2* and *HLA-DPB1* 3′ untranslated region predicted by miRanda and TargetScanHuman 7.2, respectively. BC, breast cancer; miRNA, microRNA.

Due to the high degree of sequence similarity between pseudogenes and their parent genes, pseudogenes are the “perfect bait” of their ancestral genes, crucially influencing on their parent genes by functioning as ceRNA for miRNAs or interacting with RNA-binding proteins (52), which depend on the subcellular localization of pseudogenes. To find out the underlying mechanisms of *HLA-DPB2* in BC, we predicted the distribution of *HLA-DPB2* using the IncLocator database and found that *HLA-DPB2* was mainly distributed in the cytoplasm (Figure 5A), indicating that *HLA-DPB2* regulates *HLA-DPB1* expression more likely in the ceRNA manner. As depicted in Figure 5B, after taking the intersection of the prediction results from three databases (49 miRNAs of *HLA-DPB2* from miRanda, 400 miRNAs of *HLA-DPB1* from miRWalk, and 936 miRNAs of *HLA-DPB1* from TargetScan), there were four miRNAs (*has-miR-138-5p, has-miR-370-3p, has-miR-15b-5p*, and *has-miR-330-5p*) as candidate miRNAs. We further analyzed their expression levels using the dbDEMC database. Compared with normal tissues, only *has-miR-370-3p* was downregulated (Figure 5C and Supplementary Table 4, *P* < 0.01), whereas the expressions of *has-miR-138-5p, has-miR-15b-5p*, and *has-miR-330-5p* were upregulated in BC tissues (Figure 5C). These results demonstrated that *HLA-DPB2* might serve as ceRNA to improve *HLA-DPB2* expression by sponging *has-miR-370-3p* (Figure 5D).

### *HLA-DPB2/HLA-DPB1* Axis Is Positively Associated With Immune Infiltration in Breast Cancer

**Figure 6:**
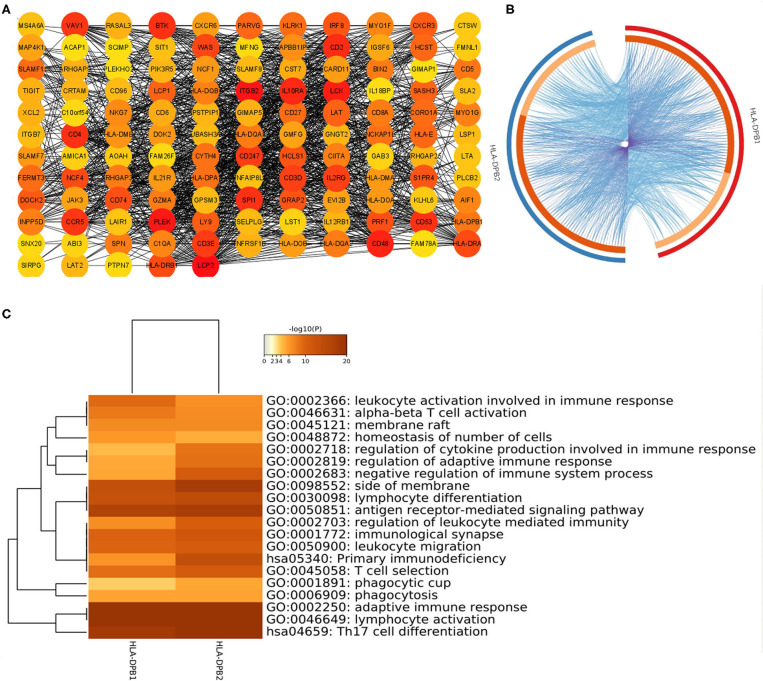
Construction of PPI network and GO enrichment analysis for top 100 co-expressed genes of *HLA-DPB2/HLA-DPB1* in BC. **(A)** The PPI network of the top 100 co-expressed genes of *HLA-DPB2* and *HLA-DPB1* visualized by Cytoscape v3.8; **(B)** Circos overlap of *HLA-DPB2* and *HLA-DPB1* co-expression genes in BC. Purple lines link the same gene that is shared by two gene lists; blue lines link the different genes where they fall into the same ontology term; **(C)** the top 20 enrichment terms of the top 100 correlated genes of *HLA-DPB2* and *HLA-DPB1*. BC, breast cancer; PPI, protein–protein interaction; GO, gene ontology.

**Figure 7:**
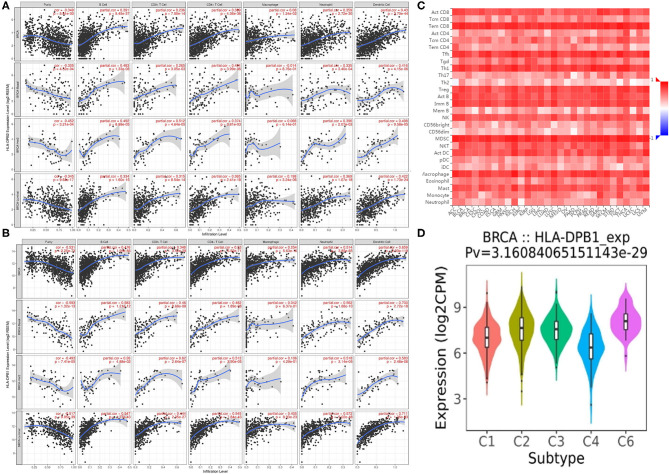
Correlation of *HLA-DPB2/HLA-DPB1* expression with immune infiltration level in BC. **(A,B)** Spearman correlation of immune infiltration level with *HLA-DPB2* and *HLA-DPB1* in BC and its subtype using TIMER database; **(C)** the heatmap of correlation between the abundance of tumor-infiltrating lymphocytes (TILs) and *HLA-DPB1* expression in BC using TISIDB database; **(D)** the distribution of *HLA-DPB1* expression across immune subtypes using TISIDB database, the statistical significance of differential expression evaluated using Kruskal–Wallis test. BC, breast cancer; C1 (wound healing); C2 (IFN-gamma dominant); C3 (inflammatory); C4 (lymphocyte depleted); C6 (TGF-b dominant).

Co-expression analysis can give us some important clues for studying the function of *HLA-DPB2*. To determine the underlying roles of the *HLA-DPB2/HLA-DPB1* axis in BC, we obtained the top 100 co-expressed genes of *HLA-DPB2* and *HLA-DPB1* using the UALCAN database, as shown in Supplementary Table 5 and Supplementary Figure 5. There was a lot of overlap between the top 100 related genes of *HLA-DPB2* and *HLA-DPB1* (Figure 6B). Then, the STRING database was applied to build a PPI network for the top 100 correlated genes of *HLA-DPB2* and *HLA-DPB1* (Figure 6A). Moreover, we performed GO and Kyoto Encyclopedia of Genes and Genomes pathway enrichment analysis of these genes and observed that *HLA-DPB2* and *HLA-DPB1* were closely concerned with immune-related biological functions (Figure 6C). Thus, the *HLA-DPB2/HLA-DPB1* axis may have something to do with the immune response against tumors. Therefore, we further investigated the association of *HLA-DPB2* and *HLA-DPB1* expression with immune infiltration abundances in BC and its subtype by using TIMER. The results showed that *HLA-DPB2* and *HLA-DPB1* expression levels have significantly positive associations with infiltrating abundances of B cells, CD8+ T cells, CD4+ T cells, neutrophils, and DCs in BRCA and its subtype (Figures 7A,B). Moreover, we further validated the relationship between the abundance of 28 TILs and *HLA-DPB1* expression in BRCA using data from the TISIDB database (Figure 7C and Supplementary Figure 6). Following the TIMER data, high *HLA-DPB1* expression strongly correlated with high infiltration abundances of activated CD8 T cells (Act CD8), effector memory CD8 T cells (Tem CD8), T-follicular helper cells (Tfh), type 1 T helper cells (Th 1), regulatory T cells (Treg), activated B cells (Act B), immature B cells (Imm B), natural killer (NK) cells, myeloid-derived suppressor cells, and macrophages in BRCA. Specially, we discovered that the mRNA level of *HLA-DPB1* was obviously lower in the lymphocyte-depleted BC immune subtype (Figure 7D). These findings demonstrate that the *HLA-DPB2/HLA-DPB1* axis may be involved in recruiting tumor-infiltrating immune cells into the tumor microenvironment.

### Correlation Analysis Between *HLA-DPB1/HLA-DPB1* Expression and the Immune Biomarkers

To ascertain the relationship between *HLA-DPB1/HLA-DPB1* and the various immune infiltrating cells, we further evaluated the associations between *HLA-DPB1/HLA-DPB1* and biomarkers of tumor-infiltrating immune cells in BC and its subtype using data from the TIMER database. It was found that the expression levels of *HLA-DPB2* and *HLA-DPB1* were obviously associated with most immune biomarkers of diverse immune cells in BRCA, BRCA-luminal, and BRCA-basal (Table 1). Moreover, we discovered that the expression levels of most biomarkers of B cell, monocytes, NK cells, dendritic cells, T cells, CD8+ T cells, Th1, T-cell exhaustion, and TAMs have strong correlations with *HLA-DPB2* and *HLA-DPB1* expressions in BRCA and its subtype (Table 1). Specifically, we showed that IRF5 of M1 macrophage, CD11b and CCR7 of neutrophils, and IL21 of Tfh significantly correlated with the *HLA-DPB2* and *HLA-DPB1* expressions in BRCA and its subtype. The correlations of the *HLA-DPB2* and *HLA-DPB1* expressions with the markers of M2 macrophage, Th2, Th17, and Treg differed among the various BC subtypes. These results showed strong relationships between *HLA-DPB2/ HLA-DPB1* and B cells, monocytes, NK cells, dendritic cells, T cells, CD8+ T cells, Th1, T-cell exhaustion, and TAM infiltration.

**Table 1:** Correlation analysis between *HLA-DPB2/HLA-DPB1* and biomarkers of immune cells in BC and its subtype (TIMER).

**Description**	**Gene markers**	***HLA-DPB2***								***HLA-DPB1***							
		**BRCA**		**BRCA-luminal**		**BRCA-Her2+**		**BRCA-basal**		**BRCA**		**BRCA-luminal**		**BRCA-Her2+**		**BRCA-basal**	
		**Cor**	***P***	**Cor**	***P***	**Cor**	***P***	**Cor**	***P***	**Cor**	***P***	**Cor**	***P***	**Cor**	***P***	**Cor**	***P***
B cell	CD19	**0.411**	9.90E-42^***^	**0.329**	3.19E-15^***^	**0.500**	6.56E-05^***^	**0.411**	9.90E-42^***^	**0.463**	7.30E-54^***^	**0.408**	2.85E-23^***^	**0.552**	7.14E-06^***^	**0.463**	7.30E-54^***^
	CD79A	**0.408**	3.88E-41^***^	**0.327**	4.55E-15^***^	**0.513**	3.83E-05^***^	**0.408**	3.88E-41^***^	**0.467**	4.47E-55^***^	**0.437**	9.03E-27^***^	**0.530**	1.91E-05^***^	**0.467**	4.47E-55^***^
Monocyte	CD86	**0.425**	6.45E-45^***^	**0.422**	6.31E-25^***^	**0.383**	3.02E-03^*^	**0.425**	6.45E-45^***^	**0.655**	4.57E-123^***^	**0.676**	3.89E-74^***^	**0.610**	3.66E-07^***^	**0.655**	4.57E-123^***^
	CD115 (CSF1R)	**0.372**	5.65E-34^***^	**0.368**	5.85E-19^***^	**0.359**	5.60E-03^*^	**0.372**	5.65E-34^***^	**0.664**	2.63E-127^***^	**0.692**	8.82E-79^***^	**0.550**	7.66E-06^***^	**0.664**	2.63E-127^***^
M1 Macrophage	INOS (NOS2)	0.046	1.43E-01	0.010	8.10E-01	0.037	7.83E-01	0.046	1.43E-01	0.058	6.73E-02	0.057	1.84E-01	0.152	2.56E-01	0.058	6.73E-02
	IRF5	**0.288**	1.76E-20^***^	**0.223**	1.39E-07^***^	**0.401**	1.80E-03^*^	**0.288**	1.76E-20^***^	**0.383**	4.14E-36^***^	**0.300**	7.80E-13^***^	**0.526**	2.24E-05^***^	**0.383**	4.14E-36^***^
	COX2(PTGS2)	**0.082**	9.29E-03^*^	0.039	3.65E-01	0.089	5.06E-01	**0.082**	9.29E-03^*^	0.035	2.69E-01	**0.123**	4.01E-03^*^	–0.044	7.41E-01	0.035	2.69E-01
M2 Macrophage	CD163	**0.228**	3.72E-13^***^	**0.215**	4.21E-07^***^	0.299	2.24E-02	**0.228**	3.72E-13^***^	**0.438**	9.42E-48^***^	**0.455**	3.09E-29^***^	**0.387**	2.72E-03^*^	**0.438**	9.42E-48^***^
	VSIG4	**0.181**	8.85E-09^***^	**0.188**	9.42E-06^***^	0.246	6.27E-02	**0.181**	8.85E-09^***^	**0.420**	8.26E-44^***^	**0.446**	5.04E-28^***^	0.188	1.56E-01	**0.420**	8.26E-44^***^
	MS4A4A	**0.324**	1.11E-25^***^	**0.332**	1.68E-15^***^	**0.393**	2.28E-03^*^	**0.324**	1.11E-25^***^	**0.552**	2.96E-80^***^	**0.536**	7.42E-42^***^	0.544	1.02E-05^***^	**0.552**	2.96E-80^***^
Neutrophils	CD66b (CEACAM8)	0.064	4.28E-02	0.026	5.49E-01	0.050	7.11E-01	0.064	4.28E-02	0.025	4.30E-01	0.017	7.01E-01	–0.063	6.39E-01	0.025	4.30E-01
	CD11b (ITGAM)	**0.332**	6.09E-27^***^	**0.320**	1.81E-14^***^	**0.352**	6.79E-03^*^	**0.332**	6.09E-27^***^	**0.532**	1.33E-73^***^	**0.544**	2.16E-43^***^	**0.493**	8.45E-05^***^	**0.532**	1.33E-73^***^
	CCR7	**0.437**	1.04E-47^***^	**0.384**	1.33E-20^***^	**0.508**	4.61E-05^***^	**0.437**	1.04E-47^***^	**0.543**	3.04E-77^***^	**0.487**	9.79E-34^***^	**0.630**	1.19E-07^***^	**0.543**	3.04E-77^***^
Natural killer cell	KIR2DL1	**0.217**	4.33E-12^***^	**0.215**	4.26E-07^***^	**0.374**	3.82E-03^*^	**0.217**	4.33E-12^***^	**0.304**	1.07E-22^***^	**0.299**	1.04E-12^***^	**0.483**	1.24E-04^**^	**0.304**	1.07E-22^***^
	KIR2DL3	**0.248**	2.20E-15^***^	**0.224**	1.34E-07^***^	**0.435**	6.40E-04^**^	**0.248**	2.20E-15^***^	**0.319**	5.25E-25^***^	**0.302**	6.00E-13^***^	**0.492**	8.62E-05^***^	**0.319**	5.25E-25^***^
	KIR2DL4	**0.312**	6.34E-24^***^	**0.271**	1.31E-10^***^	**0.468**	2.10E-04^**^	**0.312**	6.34E-24^***^	**0.350**	5.19E-30^***^	**0.321**	1.74E-14^***^	**0.610**	3.75E-07^***^	**0.350**	5.19E-30^***^
	KIR3DL1	**0.233**	9.08E-14^***^	**0.189**	9.17E-06^***^	**0.355**	6.28E-03^*^	**0.233**	9.08E-14^***^	**0.315**	2.31E-24^***^	**0.267**	2.29E-10^***^	0.295	2.48E-02	**0.315**	2.31E-24^***^
	KIR3DL2	**0.356**	3.94E-31^***^	**0.291**	4.54E-12^***^	**0.482**	1.27E-04^**^	**0.356**	3.94E-31^***^	**0.393**	4.73E-38^***^	**0.388**	5.42E-21^***^	**0.572**	2.72E-06^***^	**0.393**	4.73E-38^***^
	KIR3DL3	**0.195**	6.10E-10^***^	**0.134**	1.72E-03^*^	0.333	1.07E-02	**0.195**	6.10E-10^***^	**0.214**	9.17E-12^***^	**0.167**	8.99E-05^***^	**0.497**	7.22E-05^***^	**0.214**	9.17E-12^***^
	KIR2DS4	**0.208**	3.78E-11^***^	**0.167**	9.17E-05^***^	**0.431**	7.31E-04^**^	**0.208**	3.78E-11^***^	**0.260**	8.06E-17^***^	**0.225**	1.08E-07^***^	**0.495**	7.77E-05^***^	**0.260**	8.06E-17^***^
Dendritic cell	HLA-DPB1	**0.570**	8.08E-87^***^	**0.538**	3.80E-42^***^	**0.616**	2.68E-07^***^	**0.570**	8.08E-87^***^	**−1.000**	0.00E+00^***^	**−1.000**	NA^***^	**−1.000**	0.00E+00^***^	**−1.000**	0.00E+00^***^
	HLA-DQB1	**0.443**	5.27E-49^***^	**0.381**	2.96E-20^***^	**0.443**	4.92E-04^**^	**0.443**	5.27E-49^***^	**0.703**	3.74E-149^***^	**0.676**	5.39E-74^***^	**0.591**	1.05E-06^***^	**0.703**	3.74E-149^***^
	HLA-DRA	**0.598**	1.67E-97^***^	**0.592**	8.09E-53^***^	**0.680**	4.20E-09^***^	**0.598**	1.67E-97^***^	**0.886**	0.00E+00^***^	**0.908**	4.98E-207^***^	**0.903**	3.30E-22^***^	**0.886**	0.00E+00^***^
	HLA-DPA1	**0.528**	2.46E-72^***^	**0.533**	2.55E-41^***^	**0.638**	7.18E-08^***^	**0.528**	2.46E-72^***^	**0.903**	0.00E+00^***^	**0.921**	7.33E-224^***^	**0.895**	2.43E-21^***^	**0.903**	0.00E+00^***^
	BDCA-1 (CD1C)	**0.365**	9.02E-33^***^	**0.332**	1.84E-15^***^	**0.356**	6.02E-03^*^	**0.365**	9.02E-33^***^	**0.485**	1.07E-59^***^	**0.466**	1.09E-30^***^	**0.354**	6.44E-03^*^	**0.485**	1.07E-59^***^
	BDCA-4 (NRP1)	0.006	8.48E-01	0.038	3.80E-01	0.035	7.92E-01	0.006	8.48E-01	**0.089**	5.12E-03^*^	**0.173**	5.00E-05^***^	0.126	3.45E-01	**0.089**	5.12E-03*
	CD11c (ITGAX)	**0.437**	1.13E-47^***^	**0.416**	3.41E-24^***^	**0.499**	6.70E-05^***^	**0.437**	1.13E-47^***^	**0.643**	3.48E-117^***^	**0.650**	1.24E-66^***^	**0.586**	1.35E-06^***^	**0.643**	3.48E-117^***^
T cell	CD3D	**0.552**	2.04E-80^***^	**0.496**	4.15E-35^***^	**0.620**	2.09E-07^***^	**0.552**	2.04E-80^***^	**0.734**	1.20E-168^***^	**0.697**	1.31E-80^***^	**0.793**	1.13E-13^***^	**0.734**	1.20E-168^***^
	CD3E	**0.536**	5.41E-75^***^	**0.489**	4.56E-34^***^	**0.596**	8.07E-07^***^	**0.536**	5.41E-75^***^	**0.704**	1.21E-149^***^	**0.679**	8.46E-75^***^	**0.792**	1.38E-13^***^	**0.704**	1.21E-149^***^
	CD2	**0.527**	5.48E-72^***^	**0.484**	2.44E-33^***^	**0.590**	1.07E-06^**^	**0.527**	5.48E-72^***^	**0.699**	1.02E-146^***^	**0.677**	3.52E-74^***^	**0.800**	4.98E-14^***^	**0.699**	1.02E-146^***^
CD8+ T cell	CD8A	**0.477**	1.36E-57^***^	**0.437**	7.54E-27^***^	**0.545**	9.60E-06^***^	**0.477**	1.36E-57^***^	**0.612**	3.48E-103^***^	**0.590**	1.63E-52^***^	**0.714**	3.22E-10^***^	**0.612**	3.48E-103^***^
	CD8B	**0.480**	2.08E-58^***^	**0.459**	1.04E-29^***^	**0.534**	1.60E-05^***^	**0.480**	2.08E-58^***^	**0.567**	1.18E-85^***^	**0.596**	9.73E-54^***^	**0.723**	1.52E-10^***^	**0.567**	1.18E-85^***^
Th1	STAT1	**0.240**	1.75E-14^***^	**0.205**	1.32E-06^***^	0.210	1.13E-01	**0.240**	1.75E-14^***^	**0.262**	4.08E-17^***^	**0.294**	2.67E-12^***^	**0.397**	2.05E-03^*^	**0.262**	4.08E-17^***^
	STAT4	**0.430**	4.13E-46^***^	**0.381**	2.71E-20^***^	**0.494**	8.15E-05^**^	**0.430**	4.13E-46^***^	**0.551**	5.98E-80^***^	**0.492**	1.42E-34^***^	**0.765**	2.87E-12^***^	**0.551**	5.98E-80^***^
	TNF-α (TNF)	**0.225**	7.76E-13^***^	**0.162**	1.46E-04^**^	0.273	3.82E-02	**0.225**	7.76E-13^***^	**0.259**	9.27E-17^***^	**0.272**	1.15E-10^***^	0.314	1.64E-02	**0.259**	9.27E-17^***^
	IFN-γ (IFNG)	**0.433**	1.31E-46^***^	**0.409**	2.02E-23^***^	**0.589**	1.16E-06^***^	**0.433**	1.31E-46^***^	**0.544**	1.61E-77^***^	**0.554**	3.21E-45^***^	**0.651**	3.25E-08^***^	**0.544**	1.61E-77^***^
	T-bet (TBX21)	**0.516**	9.75E-69^***^	**0.457**	1.68E-29^***^	**0.589**	1.17E-06^***^	**0.516**	9.75E-69^***^	**0.683**	1.49E-137^***^	**0.625**	2.22E-60^***^	**0.762**	3.54E-12^***^	**0.683**	1.49E-137^***^
Th2	GATA3	**−0.203**	1.05E-10^***^	–**0.140**	1.03E-03^*^	–0.085	5.27E-01	**−0.203**	1.05E-10^***^	**−0.129**	4.82E-05^***^	**−0.209**	8.83E-07^***^	0.053	6.92E-01	**−0.129**	4.82E-05^***^
	STAT6	**0.086**	6.52E-03^*^	0.056	1.91E-01	0.044	7.43E-01	**0.086**	6.52E-03^*^	**0.159**	4.33E-07^***^	0.078	6.89E-02	0.112	4.03E-01	**0.159**	4.33E-07^***^
	STAT5A	**0.205**	7.36E-11^***^	**0.161**	1.57E-04^**^	**0.401**	1.79E-03^*^	**0.205**	7.36E-11^***^	**0.308**	2.89E-23^***^	**0.330**	2.64E-15^***^	**0.518**	3.09E-05^***^	**0.308**	2.89E-23^***^
	IL13	**0.167**	1.25E-07^***^	0.107	1.26E-02	**0.393**	2.28E-03^*^	**0.167**	1.25E-07^***^	**0.200**	2.05E-10^***^	**0.167**	9.04E-05^***^	**0.349**	7.31E-03^*^	**0.200**	2.05E-10^***^
Tfh	BCL6	–0.025	4.39E-01	–0.020	6.34E-01	–0.199	1.34E-01	–0.025	4.39E-01	0.045	1.54E-01	0.036	3.97E-01	–0.279	3.38E-02	0.045	1.54E-01
	IL21	**0.307**	4.20E-23^***^	**0.269**	1.60E-10^***^	**0.434**	6.72E-04^**^	**0.307**	4.20E-23^***^	**0.306**	5.26E-23^***^	**0.300**	9.26E-13^***^	**0.362**	5.25E-03^*^	**0.306**	5.26E-23^***^
Th17	STAT3	–0.059	6.17E-02	–0.022	6.00E-01	0.172	1.96E-01	–0.059	6.17E-02	–0.052	9.99E-02	–0.006	8.81E-01	–0.027	8.42E-01	–0.052	9.99E-02
	IL17A	**0.147**	3.22E-06^***^	0.090	3.60E-02	0.091	4.99E-01	**0.147**	3.22E-06^***^	**0.145**	4.11E-06^***^	0.069	1.06E-01	0.118	3.78E-01	**0.145**	4.11E-06^***^
Treg	FOXP3	**0.433**	1.09E-46^***^	**0.387**	5.87E-21^***^	**0.589**	1.12E-06^***^	**0.433**	1.09E-46^***^	**0.530**	3.42E-73^***^	**0.601**	7.84E-55^***^	**0.707**	5.62E-10^***^	**0.530**	3.42E-73^***^
	CCR8	**0.323**	1.22E-25^***^	**0.335**	8.55E-16^***^	0.269	4.11E-02	**0.323**	1.22E-25^***^	**0.377**	5.91E-35^***^	**0.465**	1.26E-30^***^	**0.398**	1.97E-03^*^	**0.377**	5.91E-35^***^
	STAT5B	–0.019	5.48E-01	0.010	8.10E-01	**0.349**	7.24E-03^*^	–0.019	5.48E-01	0.016	6.22E-01	0.055	2.01E-01	0.268	4.24E-02	0.016	6.22E-01
	TGFβ (TGFB1)	**0.226**	4.98E-13^***^	**0.176**	3.48E-05^***^	0.119	3.72E-01	**0.226**	4.98E-13^***^	**0.444**	2.52E-49^***^	**0.379**	4.87E-20^***^	0.286	2.98E-02	**0.444**	2.52E-49^***^
T cell exhaustion	PD-1 (PDCD1)	**0.532**	7.13E-74^***^	**0.489**	4.59E-34^***^	**0.542**	1.11E-05^***^	**0.532**	7.13E-74^***^	**0.648**	2.41E-119^***^	**0.599**	2.54E-54^***^	**0.801**	4.64E-14^***^	**0.648**	2.41E-119^***^
	CTLA4	**0.479**	3.42E-58^***^	**0.419**	1.50E-24^***^	**0.585**	1.45E-06^***^	**0.479**	3.42E-58^***^	**0.555**	2.69E-81^***^	**0.523**	1.64E-39^***^	**0.744**	2.25E-11^***^	**0.555**	2.69E-81^***^
	TIM-3 (HAVCR2)	**0.387**	6.57E-37^***^	**0.396**	6.02E-22^***^	**0.407**	1.53E-03^*^	**0.387**	6.57E-37^***^	**0.632**	3.62E-112^***^	**0.642**	1.48E-64^***^	**0.572**	2.76E-06^***^	**0.632**	3.62E-112^***^
	LAG3	**0.400**	2.08E-39^***^	**0.368**	5.88E-19^***^	**0.404**	1.68E-03^*^	**0.400**	2.08E-39^***^	**0.484**	1.56E-59^***^	**0.436**	1.15E-26^***^	**0.652**	3.05E-08^***^	**0.484**	1.56E-59^***^
	GZMB	**0.417**	5.47E-43^***^	**0.374**	1.50E-19^***^	**0.520**	2.82E-05^***^	**0.417**	5.47E-43^***^	**0.528**	2.35E-72^***^	**0.511**	1.49E-37^***^	**0.715**	2.87E-10^***^	**0.528**	2.35E-72^***^
TAM	CCL2	**0.248**	2.06E-15^***^	**0.204**	1.52E-06^***^	0.190	1.53E-01	**0.248**	2.06E-15^***^	**0.354**	9.25E-31^***^	**0.364**	1.48E-18^***^	0.306	1.93E-02	**0.354**	9.25E-31^***^
	CD68	**0.339**	3.24E-28^***^	**0.346**	9.13E-17^***^	**0.367**	4.59E-03^*^	**0.339**	3.24E-28^***^	**0.584**	5.26E-92^***^	**0.610**	6.43E-57^***^	**0.450**	3.97E-04^**^	**0.584**	5.26E-92^***^
	IL10	**0.283**	9.30E-20^***^	**0.250**	3.31E-09^***^	**0.363**	5.11E-03^*^	**0.283**	9.30E-20^***^	**0.432**	2.29E-46^***^	**0.412**	1.06E-23^***^	**0.515**	3.55E-05^***^	**0.432**	2.29E-46^***^

* *P < 0.01*,

** *P < 0.001*,

*** *P < 0.0001*.

*Cor, correlation adjusted by purity; PD-1, programmed cell death protein 1; BRCA, invasive breast carcinoma*.

*The bold values indicate that the results are statistically significant*.

### Relationship Between *HLA-DPB1/HLA-DPB1* and the Immune Checkpoints in Breast Cancer

**Figure 8:**
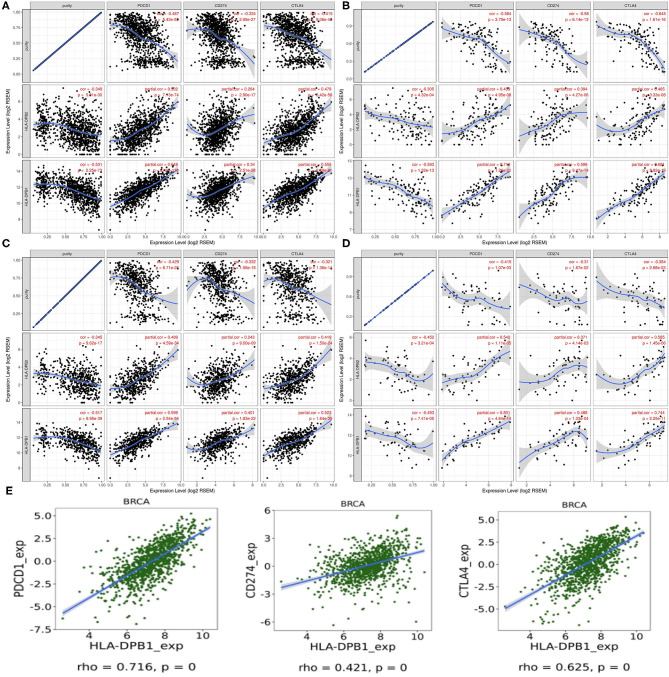
Correlation of *HLA-DPB2/HLA-DPB1* expression with PD-1, PD-L1, and CTLA-4 expression in BC. **(A–D)** Spearman correlation of *HLA-DPB2* and *HLA-DPB1* expression with expression of PD-1, PD-L1, and CTLA-4 in BRCA, BRCA-basal, BRCA-luminal, and BRCA-HER2 using TIMER, respectively. The correlation adjusted by purity. **(E)** Spearman correlation of *HLA-DPB1* expression with expression of PD-1, PD-L1, and CTLA-4 in BC using TISIDB database. BC, breast cancer; PD-1, programmed cell death protein 1; PD-L1, programmed cell death ligand 1; CTLA-4, cytotoxic T-lymphocyte-associated antigen-4; BRCA, invasive breast carcinoma.

PD-1/PD-L1 and CTLA-4 are essential molecules for tumors to escape from the immune system. Thus, we examined their relationships with *HLA-DPB1/HLA-DPB1* expression in BRCA and its molecular subtypes using data from TIMER and found that increased *HLA-DPB2* and *HLA-DPB1* expressions were strongly related with high PD-1 and CTLA-4 expressions levels and weakly associated with high PD-L1 expression levels in BRCA and its subtypes (Figures 8A–D). Similarly to TIMER data analysis, the TISIDB data analysis also revealed that *HLA-DPB1* expression positively correlated with the PD-1, PD-L1, and CTLA-4 expressions in BRCA (Figure 8E). Therefore, these results suggest that the *HLA-DPB2/HLA-DPB1* axis might serve as a useful adjunct to ICIs in the treatment of BC.

## Discussion

Previously, pseudogenes were deemed nonfunctional genes or junk genes. Nevertheless, an increasing number of research have demonstrated that pseudogenes can control functional genes through various mechanisms, thus regulating diverse physiological and pathological processes, including carcinogenesis (16, 51). Previous studies reported many tumor-related pseudogenes such as *PTENP1* (53, 54), *DUXAP10* (55), *SUMO1P3* (56), *PDIA3P* (57), *PTTG3P* (24), and *DUXAP8* (58). In the present study, we first identified 632 differentially expressed pseudogenes in BC based on data from the dreamBase database. Then, the UALCAN database was used to screen for differentially expressed pseudogenes associated with prognosis in BC for further explorations. *HLA-DPB2* was selected because it had good prognostic value and unknown biological functions in BC based on our comprehensive analysis and literature review.

Firstly, it was found that the mRNA expression of *HLA-DPB2* in BC tissues was obviously higher than that in normal breast tissues. Besides, we observed that high *HLA-DPB2* expression level was associated with ER-negative, progesterone receptor-negative, p53-mutated status, higher Scarff–Bloom–Richardson grade, basal-like, and TNBC status, and better OS. Subsequently, we ascertained the relationship between the pseudogene *HLA-DPB2* and its parent gene *HLA-DPB1* and found that *HLA-DPB1* expression strongly positively correlated with *HLA-DPB2* expression. Compared with that in normal breast samples, the mRNA expression of *HLA-DPB1* also increased in BC tissues, especially in the ER-negative, basal-like, and TNBC status. The survival analysis revealed that high *HLA-DPB1* expression levels predicted better OS, RFS, and DMFS. Moreover, multivariate analysis indicated *HLA-DPB2* and *HLA-DPB1* were independent prognostic factors for longer OS of BC patients. The earlier discussed findings indicate that *HLA-DPB2* and *HLA-DPB1* take part in the tumor suppression processes of BC and that overexpression of *HLA-DPB2* may predict a favorable prognosis by regulating the parent gene *HLA-DPB1* expression. However, the mechanism thereby *HLA-DPB2* regulates *HLA-DPB1* expression remains unknown.

Previous studies demonstrated that as lncRNAs, pseudogenes could play their roles in DNA, RNA, and protein levels through various mechanisms involving antisense RNAs, interference RNAs, and ceRNAs or a combination with RNA-binding protein, to affect their parental genes or other gene expressions (17, 50). The subcellular localization of pseudogenes determines their regulatory mechanisms. We next predicted the subcellular localization of *HLA-DPB2* using the IncLocator database and found that *HLA-DPB2* was mainly distributed in the cytoplasm. Therefore, we speculated that *HLA-DPB2* might be likely to regulate the expression of its parental gene through the ceRNA mechanism, and the prediction result suggested that *HLA-DPB2* may act as an endogenous sponge for *has-miR-370-3p* to prevent it from binding to *HLA-DPB1*. We also observed that the *has-miR-370-3p* expression was clearly downregulated in the BC samples as compared with the normal samples. Functional enrichment analysis revealed that the top 100 correlated genes of *HLA-DPB2* and *HLA-DPB1* were mainly enriched in adaptive immune response (GO: 0002250), lymphocyte activation (GO: 0046649), Th17 cell differentiation (hsa04659), alpha-beta T cell activation (GO: 0046631), leukocyte activation involved in immune response (GO: 0002366), and regulation of leukocyte mediated immunity (GO: 0002703). Therefore, we supposed that the *HLA-DPB2/HLA-DPB1* axis might exert its roles in BC by involving an immune response in the tumor microenvironment.

Some researchers have established that tumor-infiltrating immune cells can affect prognosis and the efficacies of chemoradiotherapy or immunotherapy (59–61). Thus, we examined the correlation of the *HLA-DPB2/HLA-DPB1* axis with the immune infiltration levels in BC and its subtype using data from the TIMER and TISIDB databases. The results showed that the *HLA-DPB2* and *HLA-DPB1* expression levels have obviously positive associations with the infiltrating abundances of B cells, CD8+ T cells, CD4+ T cells, neutrophils, DCs, Tfh, Th1, macrophages, and NK cells, and with the expression of most biomarkers of B cells, monocytes, NK cells, DCs, T cells, CD8+ T cells, Th1, T-cell exhaustion, and TAMs in BC and its subtype. Specially, we observed the *HLA-DPB1* expression obviously decreased in lymphocytes depleted of the BC immune subtype. Previous researchers found that the *HLA-DPB1* protein binds with *HLA-DPA1* and forms an antigen-binding complex, which can display foreign peptides to the immune system and initiate the body's immune response to attack the invading viruses or bacteria (62). *HLA-DPB1* is generally expressed in B lymphocytes, DCs, and macrophages, which can explain the strong correlation of *HLA-DPB1* expression with infiltrating levels of B cells, DCs, and macrophages in our results (63). It has been reported that *HLA-DPB1*-specific CD4+ T-cell clones can identify and dissolve myeloid and lymphoid malignanT cells expressing *HLA-DP*, which is following our results (29). Based on literature reports and our findings, we speculate that the *HLA-DPB2/HLA-DPB1* axis may convert immunologically “cold” tumors to “hot” tumors and thus exert an anticancer role in BC by recruiting T lymphocytes and NK cells into the tumor microenvironment.

Also, the continuous anti-tumor effect of ICIs not only requires sufficient lymphocytes infiltrating in the tumor microenvironment but also depends on the high immune checkpoint expression levels of tumor cells (5, 13). Hence, we also analyzed the relationship between *HLA-DPB2/HLA-DPB1* expression and immune checkpoints. Our results suggested that increased *HLA-DPB1* and *HLA-DPB1* expression levels strongly correlated with high PD-1 and CTLA-4 expression levels and weakly correlated with high PD-L1 expression levels in BC and its subtypes. These findings indicate that targeting the *HLA-DPB2/HLA-DPB1* axis might serve as a useful adjunct to ICIs in the treatment of BC.

In summary, our results suggest that the pseudogene *HLA-DPB2* may act as an endogenous RNA to adsorb *has-miR-370-3p* and upregulating its parental gene *HLA-DPB1*, thereby recruiting more TILs into the tumor microenvironment and increasing the expression of PD-1, PD-L1, and CTLA-4 in BC tissues, ultimately improving the prognosis of BC patients. More importantly, the present study may also provide a new clue to the direction of future immunotherapy for patients with BC and optimize the current immunotherapy. More laboratory research and animal trials are needed in the future to validate the findings of this study.

## Data Availability Statement

All datasets generated for this study are included in the article/Supplementary Material.

## Author Contributions

ZD and LL conceived and designed the study. LL, YZ, YW, PX, SY, YD, and DZ collected and analyzed the data. LL, YZ, JY, and MW wrote the original draft. SW, JL, FG, and ZD reviewed and edited the manuscript. All authors contributed to the article and approved the submitted version.

## Conflict of Interest

The authors declare that the research was conducted in the absence of any commercial or financial relationships that could be construed as a potential conflict of interest.

## Supplementary Material

Supplementary Figure 1 The mRNA expression level and prognostic value of *HLA-DPB2* in TCGA samples of BC using UALCAN database. BC, breast cancer.

Supplementary Figure 2 Pseudogene *HLA-DPB2* and its parental gene *HLA-DPB1* expression in three datasets of ONCOMINE databases. **(A–C)** Compared to normal breast samples, the expression level of HLA-DPB2 is higher in Finak BC and two TCGA BC, respectively; **(D–F)** Compared to normal breast samples, the expression level of HLA-DPB1 is higher in Finak BC, Karnoub BC, and Curtis BC, respectively. BC, breast cancer.

Supplementary Figure 3 The prognostic value of *HLA-DPB1* in BC patients using microarray data of Kaplan–Meier Plotter database. **(A1–D1)** The association of *HLA-DPB1* (probe: 244485_at) with OS, RFS, DMFS, and PPS in BC patients, respectively; **(A2–D2)** High expression of *HLA-DPB1* (probe: 201137_s_at) indicate better OS, RFS, DMFS, and PPS in BC patients, respectively. BC, breast cancer; OS, overall survival; RFS, relapse-free survival; DMFS, distant metastases-free survival; PPS, post-progression survival.

Supplementary Figure 4 The Forest plots of multivariate analysis of the correlation of expression of *HLA-DPB2* and *HLA-DPB1* with overall survival among BC patients. BC, breast cancer.

Supplementary Figure 5 The heatmap of top 100 correlated genes of *HLA-DPB2* and *HLA-DPB1* in BC obtained from UALCAN database. BC, breast cancer.

Supplementary Figure 6 Spearman correlation between abundance of 28 TILs and *HLA-DPB1* expression in BC using TISIDB database. BC, breast cancer.

Supplementary Figure 7 The effect of combing *HLA-DPB2* with *HLA-DPB1* expression on patient overall survival of BC using RNA-seq data downloaded from TCGA. BC, breast cancer.

Supplementary Table 1 Dysregulated pseudogenes in BC downloaded from dreamBase.

Supplementary Table 2 Dysregulated pseudogenes in BC using UALCAN database.

Supplementary Table 3 Univariate analysis and multivariate analysis of the correlation of expression of HLA-DPB2 and HLA-DPB1 with overall survival among breast cancer patients. Bold values indicate *P* < 0.05, HR, hazard ratio; CI, confidence interval.

Supplementary Table 4 Significant changes of has-miR-370-3p expression between breast cancer and normal tissues (dbDEMC 2.0). adj, adjust.

Supplementary Table 5 The top 100 correlated genes of HLA-DPB2 and HLA-DPB1 obtained from UALCAN database. CC, correlation coefficient.
