# Supplementary figures and images for: Overexpressed Pseudogene HLA-DPB2 Promotes Tumor Immune Infiltrates by Regulating HLA-DPB1 and Indicates a Better Prognosis in Breast Cancer
Source: Front Oncol. 2020 Aug 7;10:1245. doi: 10.3389/fonc.2020.01245 (PMC7438735; doi:10.3389/fonc.2020.01245)

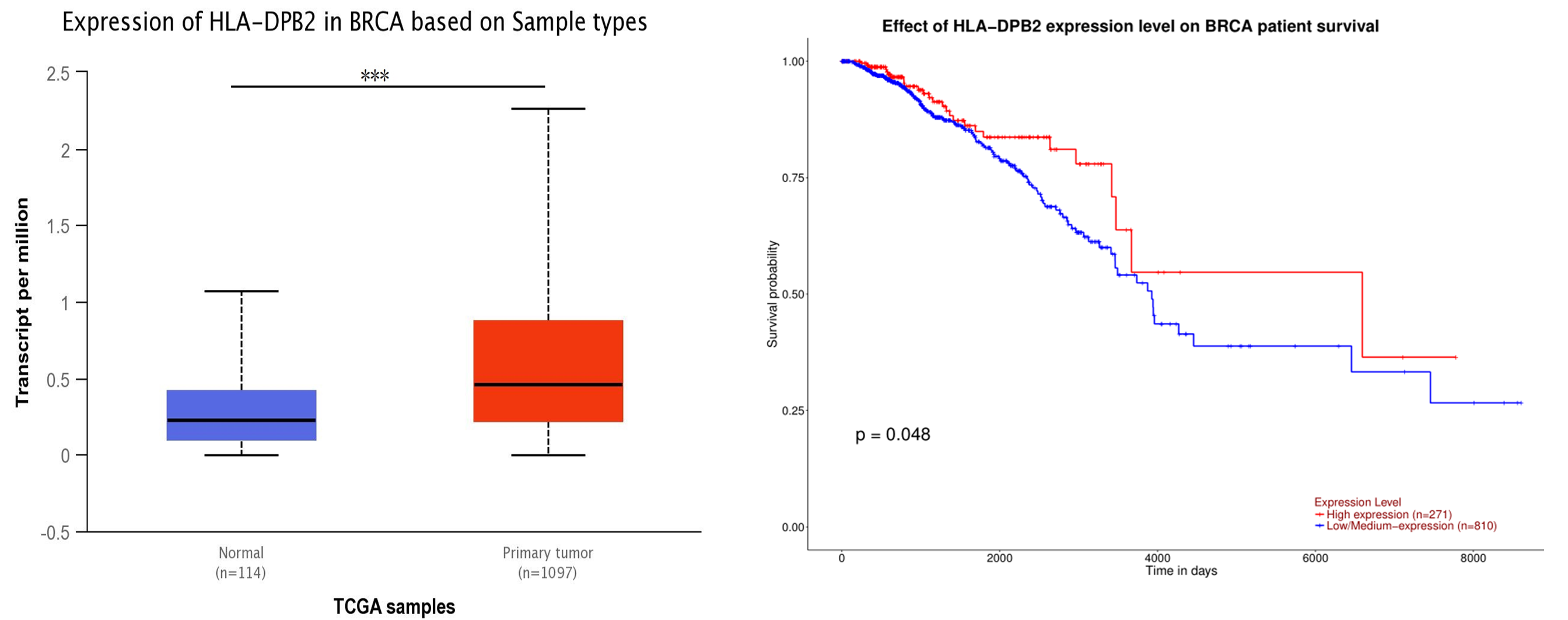

Supplement: Supplementary Figure 1 — The mRNA expression level and prognostic value of HLA-DPB2 in TCGA samples of BC using UALCAN database. BC, breast cancer. [file Image_1.TIF]

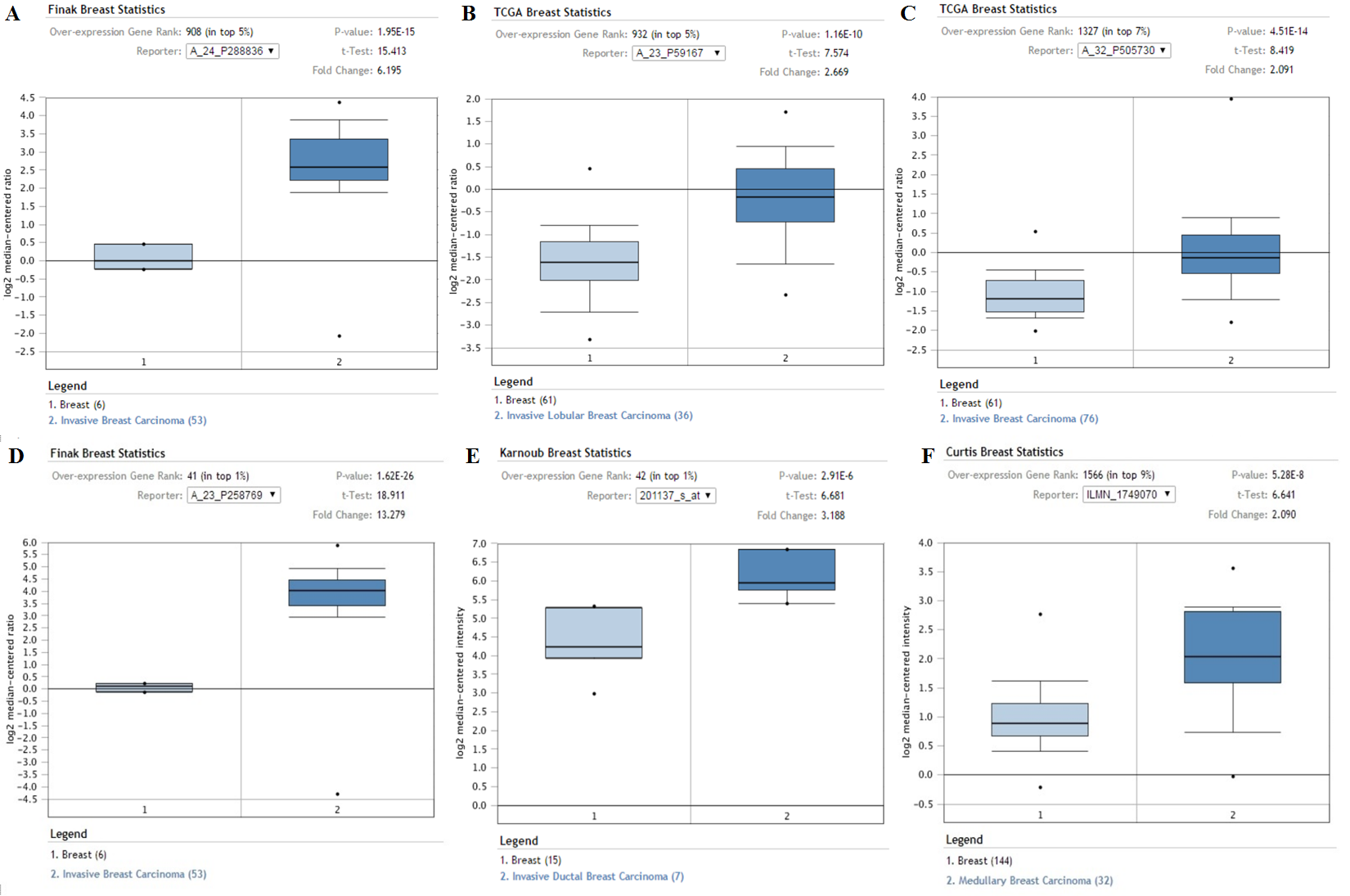

Supplement: Supplementary Figure 2 — Pseudogene HLA-DPB2 and its parental gene HLA-DPB1 expression in three datasets of ONCOMINE databases. (A–C) Compared to normal breast samples, the expression level of HLA-DPB2 is higher in Finak BC and two TCGA BC, respectively; (D–F) Compared to normal breast samples, the expression level of HLA-DPB1 is higher in Finak BC, Karnoub BC, and Curtis BC, respectively. BC, breast cancer. [file Image_2.TIF]

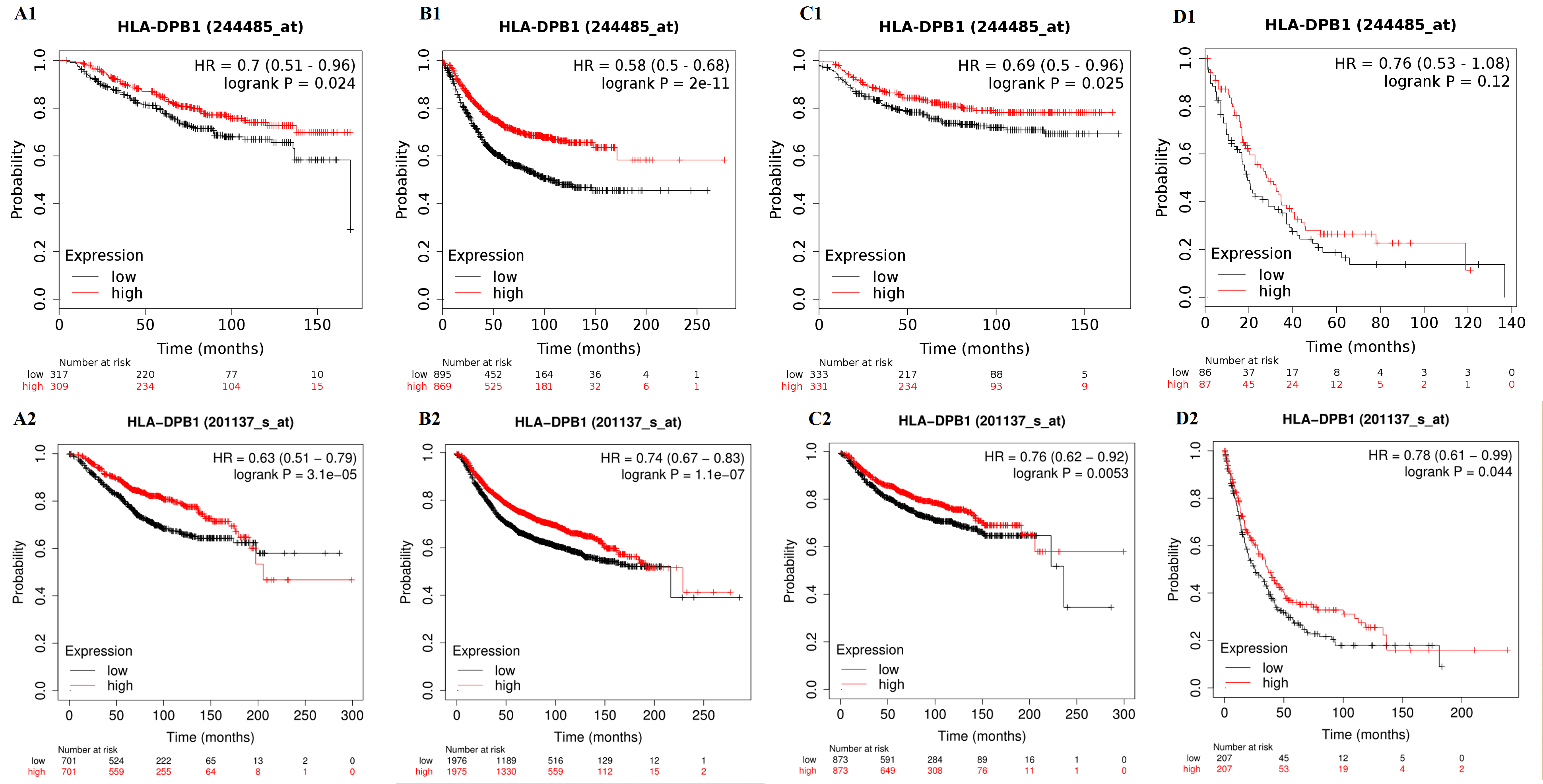

Supplement: Supplementary Figure 3 — The prognostic value of HLA-DPB1 in BC patients using microarray data of Kaplan–Meier Plotter database. (A1–D1) The association of HLA-DPB1 (probe: 244485_at) with OS, RFS, DMFS, and PPS in BC patients, respectively; (A2–D2) High expression of HLA-DPB1 (probe: 201137_s_at) indicate better OS, RFS, DMFS, and PPS in BC patients, respectively. BC, breast cancer; OS, overall survival; RFS, relapse-free survival; DMFS, distant metastases-free survival; PPS, post-progression survival. [file Image_3.TIF]

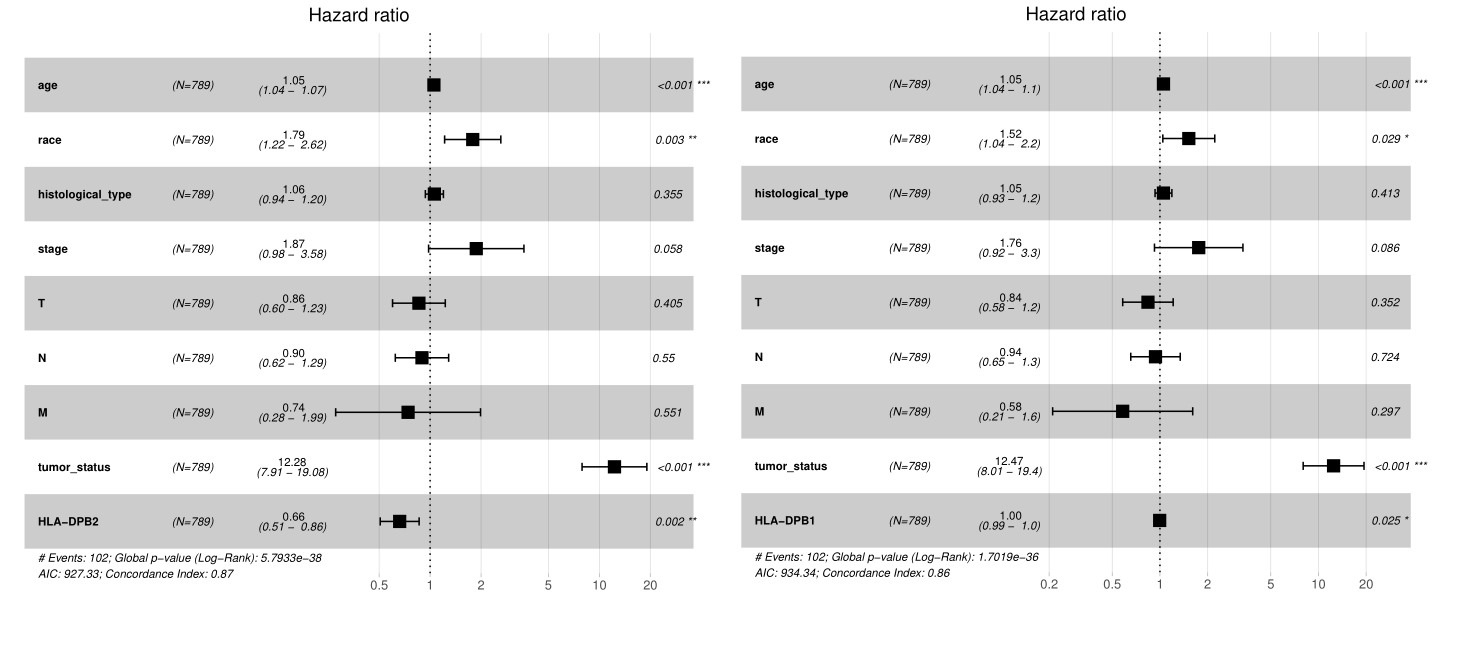

Supplement: Supplementary Figure 4 — The Forest plots of multivariate analysis of the correlation of expression of HLA-DPB2 and HLA-DPB1 with overall survival among BC patients. BC, breast cancer. [file Image_4.TIF]

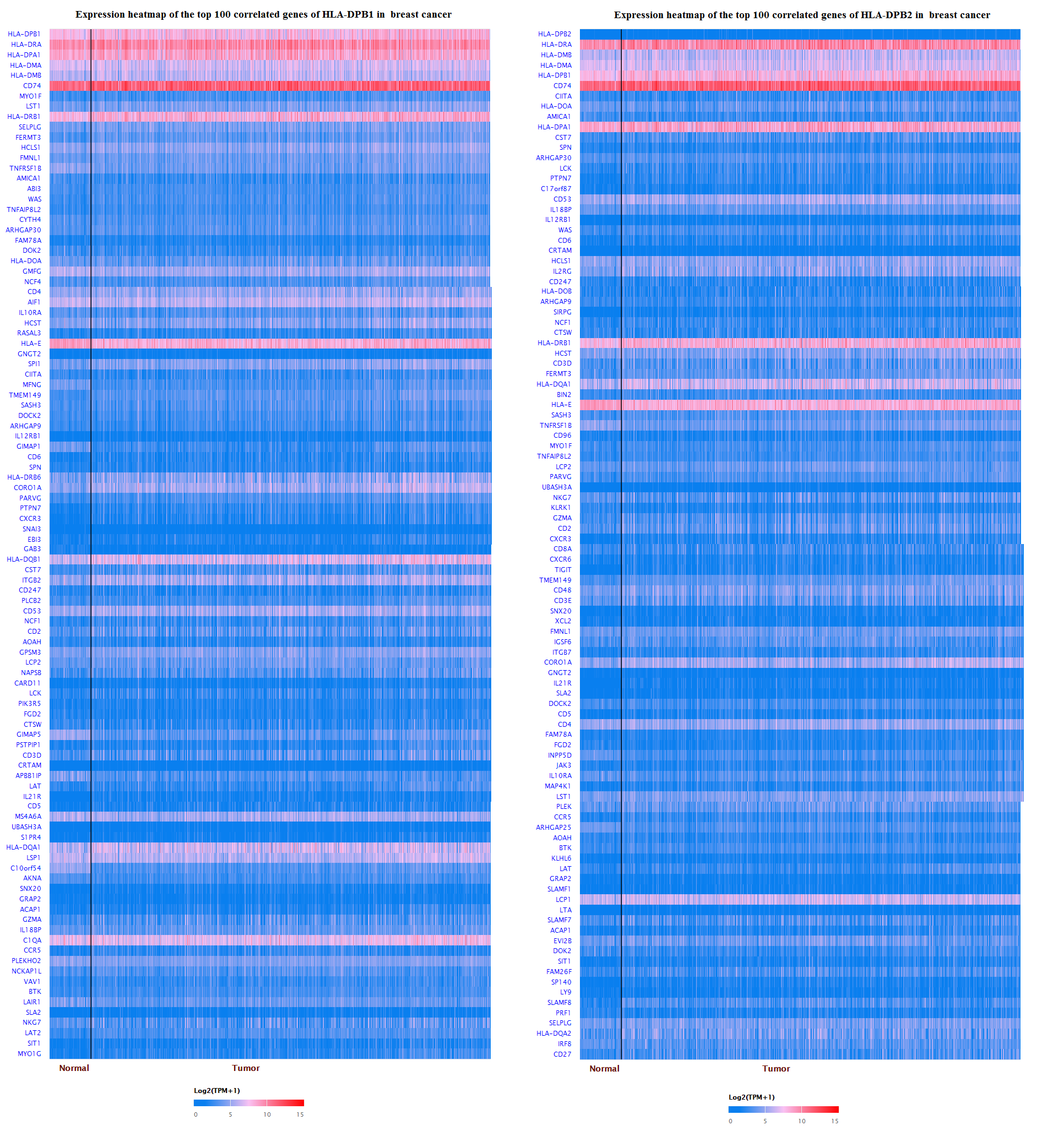

Supplement: Supplementary Figure 5 — The heatmap of top 100 correlated genes of HLA-DPB2 and HLA-DPB1 in BC obtained from UALCAN database. BC, breast cancer. [file Image_5.TIF]

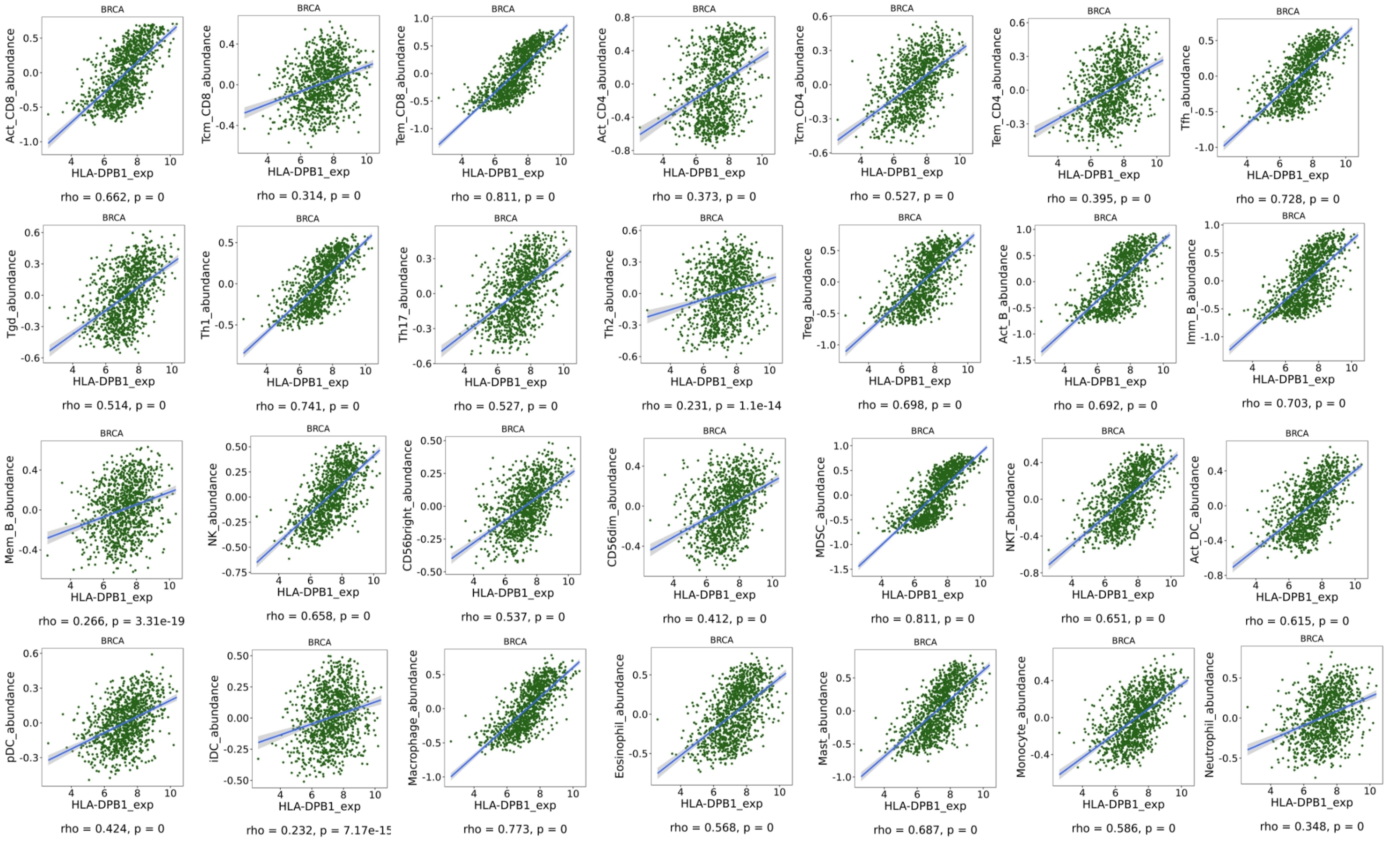

Supplement: Supplementary Figure 6 — Spearman correlation between abundance of 28 TILs and HLA-DPB1 expression in BC using TISIDB database. BC, breast cancer. [file Image_6.TIF]

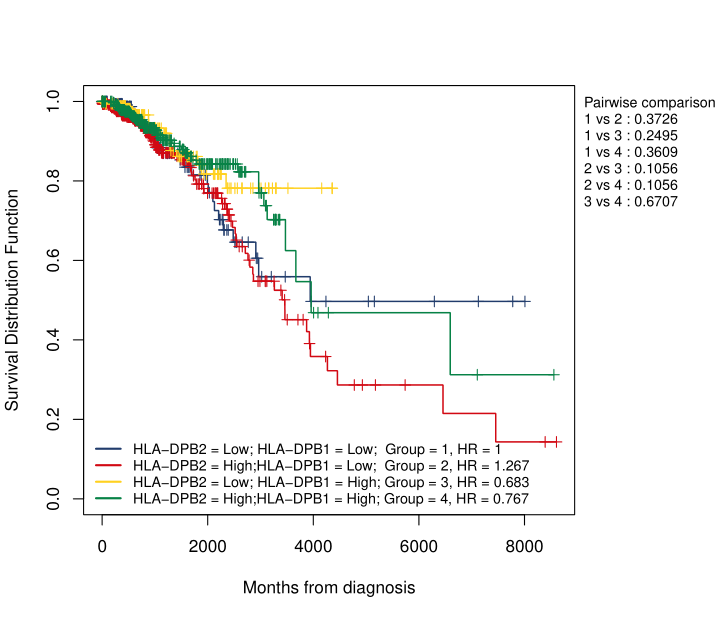

Supplement: Supplementary Figure 7 — The effect of combing HLA-DPB2 with HLA-DPB1 expression on patient overall survival of BC using RNA-seq data downloaded from TCGA. BC, breast cancer. [file Image_7.PNG]
