# Supplementary material for: Overexpressed Pseudogene HLA-DPB2 Promotes Tumor Immune Infiltrates by Regulating HLA-DPB1 and Indicates a Better Prognosis in Breast Cancer
Source: Front Oncol. 2020 Aug 7;10:1245. doi: 10.3389/fonc.2020.01245 (PMC7438735; doi:10.3389/fonc.2020.01245)
Supplement: Supplementary Table 1 — Dysregulated pseudogenes in BC downloaded from dreamBase. [file Data_Sheet_1.docx]

**Supplementary Table 1.** Dysregulated pseudogenes in BC downloaded from dreamBase.

| GeneName | Fold Change | GeneName | Fold Change |
| --- | --- | --- | --- |
| AL162151.3 | 1871.5268 | RP11-258C19.4 | -54.1917 |
| HIST1H1PS1 | 178.52719 | RP11-848P1.7 | -47.17661 |
| STK19B | 64 | RP11-574E24.3 | -42.51795 |
| SNRPFP1 | 39.124489 | FP325317.1 | -35.26096 |
| AC092569.2 | 37.014022 | MT1JP | -21.25897 |
| SPATA20P1 | 35.017398 | UBE2Q2P6 | -20.67765 |
| CH507-42P11.2 | 32 | AC129778.2 | -19.29293 |
| SUMO1P4 | 22.008669 | RP11-386I14.2 | -16.67945 |
| CTD-2528L19.3 | 16 | CETN4P | -13.08643 |
| KLKP1 | 15.032364 | RP11-115D7.3 | -13.08643 |
| CTD-3088G3.4 | 15.032364 | CYP4F24P | -13.08643 |
| RP11-382D8.3 | 14.025692 | LOC100421166 | -12.64066 |
| TPSP2 | 12.996038 | EGFEM1P | -12.295 |
| RP11-895M11.2 | 12.041974 | CES1P1 | -12.04197 |
| MRPL40P1 | 12.041974 | OR2S1P | -11.47164 |
| Z69890.1 | 11.004335 | CNTNAP3P2 | -11.00433 |
| CYCSP24 | 11.004335 | RP11-381O7.3 | -11.00433 |
| LGALS17A | 11.004335 | RP11-350D17.3 | -10.70342 |
| RP11-613E4.5 | 11.004335 | RP11-459D22.1 | -10.05611 |
| RP11-424C20.2 | 11.004335 | RP11-467L19.11 | -9.063071 |
| RP11-247I13.3 | 11.004335 | RP4-673D20.3 | -8.876556 |
| RP1-34B20.4 | 10.852835 | RP11-481H12.1 | -8.633826 |
| HNRNPA1P21 | 10.338823 | bP-2189O9.2 | -7.835362 |
| PTMAP1 | 9.9866444 | OR7E13P | -7.727491 |
| RP11-529H20.3 | 9.9866444 | OR7E154P | -7.568461 |
| RP11-719K4.3 | 9.9866444 | TRIM60P17 | -7.412704 |
| SLC25A1P5 | 9.0004679 | AOC4P | -7.412704 |
| OR7E62P | 9.0004679 | CTD-2104P17.1 | -6.964405 |
| TRMT112P6 | 9.0004679 | ANKRD20A17P | -6.821079 |
| RP4-765C7.2 | 9.0004679 | PSAT1P3 | -6.821079 |
| RPS26P11 | 9.0004679 | PRADC1P1 | -6.543216 |
| RPS20P4 | 8 | CTD-2262B20.1 | -6.543216 |
| RP1-13D10.3 | 8 | RP11-193F5.4 | -6.408559 |
| NT5CP2 | 8 | RP11-589M4.4 | -6.32033 |
| TMED10P1 | 8 | RPL21P135 | -6.233317 |
| GTF2IP23 | 8 | HMGB3P10 | -6.19026 |
| RP11-161H23.10 | 8 | CPHL1P | -6.147501 |
| RP11-378J18.6 | 7.0128458 | bP-21264C1.1 | -6.020987 |
| NSFP1 | 7.0128458 | CSPG4P5 | -5.938094 |
| CTA-313A17.3 | 7.0128458 | PTGES3P2 | -5.938094 |
| RP4-612B18.3 | 7.0128458 | RPSAP53 | -5.938094 |
| RP11-430K21.2 | 7.0128458 | RP11-241F15.1 | -5.897077 |
| RP11-1012A1.7 | 7.0128458 | AADACP1 | -5.897077 |
| HNRNPA1P57 | 7.0128458 | KRT16P6 | -5.897077 |
| PPIAP6 | 7.0128458 | BET1P1 | -5.775717 |
| CBX3P4 | 7.0128458 | RP11-311D14.1 | -5.735821 |
| CYP2B7P | 6.9162979 | OR7E94P | -5.656854 |
| RPLP0P2 | 6.1050368 | RP11-182J1.14 | -5.656854 |
| PPIAP2 | 6.020987 | RP11-460N11.2 | -5.656854 |
| RP11-353N4.5 | 6.020987 | CTD-2501B8.5 | -5.656854 |
| RP4-803A2.1 | 6.020987 | SULT1C2P1 | -5.656854 |
| RPL18P10 | 6.020987 | RPL21P10 | -5.656854 |
| GAPDHP22 | 6.020987 | RP11-405L18.4 | -5.656854 |
| CARD17 | 6.020987 | PKD1L2 | -5.656854 |
| RP11-264F23.1 | 6.020987 | AC005682.6 | -5.388934 |
| RP11-486A14.1 | 5.2415736 | TPTEP1 | -5.205367 |
| AC090804.1 | 4.9933222 | CASP12 | -5.063026 |
| PPIAP19 | 4.9933222 | GGT3P | -5.063026 |
| RP11-288G3.4 | 4.9933222 | VN1R85P | -4.993322 |
| BNIP3P24 | 4.9933222 | ZNF300P1 | -4.993322 |
| FAM201B | 4.9933222 | RPL23AP49 | -4.924578 |
| AP000662.9 | 4.9933222 | TUBB2BP1 | -4.756828 |
| PPIAP13 | 4.9933222 | GGTA1P | -4.756828 |
| RPL36P4 | 4.9933222 | ADGRF5P2 | -4.756828 |
| TREML3P | 4.9933222 | RP1-34L19.1 | -4.756828 |
| AC068137.13 | 4.9933222 | AC060834.2 | -4.756828 |
| AL590762.7 | 4.9933222 | FRMPD2B | -4.756828 |
| HIST2H2BC | 4.6913398 | UBE2Q2L | -4.626753 |
| UBE2SP2 | 4.5630549 | HIGD1AP11 | -4.594793 |
| UBE2SP1 | 4.5630549 | RP11-231P20.2 | -4.594793 |
| BCRP7 | 4.5002339 | USP32P1 | -4.563055 |
| KRT8P7 | 4.5002339 | RPL23AP1 | -4.500234 |
| ATP5G1P4 | 4.4691486 | SLC2A3P2 | -4.469149 |
| RP11-480I12.5 | 4.2574807 | CTBP2P8 | -4.469149 |
| RP5-1065J22.4 | 4 | RP11-748H22.1 | -4.40762 |
| TPM3P8 | 4 | LINC00982 | -4.40762 |
| RPS3AP2 | 4 | PABPC1P4 | -4.377175 |
| AC006539.3 | 4 | CDC20P1 | -4.346939 |
| BTBD10P2 | 4 | CTD-2311M21.2 | -4.316913 |
| RP11-58A17.3 | 4 | ZNF209P | -4.316913 |
| RP11-108F13.2 | 4 | NMD3P1 | -4.316913 |
| ALG1L15P | 4 | IFNWP19 | -4.257481 |
| RP11-889L3.4 | 4 | CTD-2224J9.8 | -4.257481 |
| RP11-146E23.2 | 4 | RP4-631H13.6 | -4.257481 |
| KRT8P8 | 4 | RPL7AP28 | -4.084049 |
| AC009961.2 | 4 | OR7E22P | -4.027822 |
| RP4-539M6.22 | 4 | MT1L | -4.027822 |
| DPH3P1 | 4 | ZNF204P | -4 |
| RP11-810P12.1 | 4 | MAGOH2P | -3.97237 |
| B3GAT3P1 | 4 | RP11-835E18.4 | -3.89062 |
| AC090286.2 | 4 | HNRNPA1P33 | -3.837056 |
| FAM172BP | 4 | ST13P10 | -3.837056 |
| AC002310.10 | 4 | RP4-814D15.1 | -3.837056 |
| PIGFP1 | 4 | SEC1P | -3.837056 |
| CDIPT-AS1 | 3.758091 | RP11-446H18.1 | -3.837056 |
| CXCR2P1 | 3.758091 | WDR45BP1 | -3.837056 |
| RP11-433A19.2 | 3.6807506 | RP11-779O18.2 | -3.837056 |
| OACYLP | 3.5064229 | RP11-44D5.1 | -3.837056 |
| HIST2H2BD | 3.4822023 | OR7E12P | -3.837056 |
| RP11-480I12.9 | 3.4105396 | GAPDHP32 | -3.837056 |
| KRT8P48 | 3.4105396 | AGGF1P3 | -3.837056 |
| CYP21A1P | 3.3172782 | RP11-64D22.1 | -3.837056 |
| GUCY1B2 | 3.2490096 | CH507-210P18.3 | -3.837056 |
| SLC25A24P1 | 3.2490096 | RP11-54D18.2 | -3.758091 |
| SORD2P | 3.226567 | TIMM8AP1 | -3.732132 |
| STMN1P1 | 3.1166583 | RP11-693N9.2 | -3.655326 |
| KRT8P45 | 3.09513 | UBE2CP2 | -3.605002 |
| GAPDHP60 | 3.0104935 | TSSC2 | -3.5801 |
| RP11-345J13.1 | 3.0104935 | RP11-111F5.2 | -3.5801 |
| KRT18P10 | 3.0104935 | RPSAP70 | -3.555371 |
| RP11-332L8.1 | 3.0104935 | RP11-369K16.1 | -3.530812 |
| MED15P9 | 3.0104935 | DNM1P51 | -3.530812 |
| RP1-315G1.1 | 3.0104935 | FTOP1 | -3.530812 |
| RP11-95M15.2 | 3.0104935 | FAM106CP | -3.530812 |
| RP11-1148O4.1 | 3.0104935 | RP11-460E7.8 | -3.506423 |
| RPL7P24 | 3.0104935 | TNXA | -3.482202 |
| BRI3BPP1 | 3.0104935 | RP11-730A19.5 | -3.482202 |
| RP11-19G24.1 | 3.0104935 | RP11-89K11.1 | -3.41054 |
| RP11-309L24.6 | 3.0104935 | LINC00933 | -3.41054 |
| VN1R5 | 3.0104935 | RP11-284B18.3 | -3.386981 |
| MYO5BP2 | 3.0104935 | AC136289.1 | -3.386981 |
| KRT18P48 | 3.0104935 | WHAMMP2 | -3.340352 |
| RNF2P1 | 3.0104935 | ZNF833P | -3.340352 |
| RPSAP31 | 3.0104935 | AC011330.5 | -3.340352 |
| RP11-183G22.1 | 3.0104935 | FAM96AP2 | -3.340352 |
| MRPL3P1 | 3.0104935 | RRN3P1 | -3.340352 |
| KRT19P1 | 3.0104935 | RP11-777F6.3 | -3.317278 |
| CTD-2013N17.1 | 3.0104935 | AC132008.1 | -3.317278 |
| RPF2P1 | 3.0104935 | BX842568.2 | -3.294364 |
| ABCC13 | 3.0104935 | MRPL35P2 | -3.294364 |
| HNRNPA3P3 | 3.0104935 | GOLGA6L5P | -3.226567 |
| AC013439.4 | 3.0104935 | NDUFAF4P3 | -3.226567 |
| RPL7P22 | 3.0104935 | RPL7P18 | -3.226567 |
| TERF1P5 | 3.0104935 | GOLGA8IP | -3.226567 |
| LINC00634 | 3.0104935 | MTCYBP21 | -3.226567 |
| KRT18P55 | 3.0104935 | RP11-96C23.11 | -3.20428 |
| AC020915.2 | 3.0104935 | VN1R20P | -3.138336 |
| HNRNPA3P1 | 3.0104935 | FER1L5 | -3.138336 |
| OR2W6P | 3.0104935 | CH17-472G23.1 | -3.138336 |
| TUBB8P8 | 3.0104935 | HIST2H2BA | -3.116658 |
| FDPSP8 | 3.0104935 | LRRC37A7P | -3.09513 |
| RP11-214J9.1 | 3.0104935 | HERC2P3 | -3.09513 |
| HMGA1P8 | 2.907945 | CECR7 | -3.09513 |
| H2AFZP3 | 2.8088898 | A2MP1 | -3.07375 |
| KRT8P3 | 2.7320805 | BMS1P11 | -3.07375 |
| VN1R48P | 2.6758551 | GGTLC5P | -3.07375 |
| OR7E91P | 2.6207868 | AC008280.3 | -3.010493 |
| POTEKP | 2.6026837 | BMS1P7 | -3.010493 |
| PPP1R14BP3 | 2.5491213 | UBE2Q2P1 | -2.989698 |
| RP11-564D11.3 | 2.5491213 | VWFP1 | -2.989698 |
| AZGP1P1 | 2.5315132 | ATP5F1P5 | -2.969047 |
| IL9RP3 | 2.5140267 | RP11-454L9.2 | -2.948538 |
| WASF5P | 2.5140267 | MARK2P8 | -2.907945 |
| KRT18P28 | 2.5140267 | AP000344.4 | -2.907945 |
| LRRC37A9P | 2.5140267 | GAPDHP42 | -2.907945 |
| AC064850.4 | 2.5140267 | NBEAP1 | -2.907945 |
| AC013268.3 | 2.5140267 | RP11-12A2.1 | -2.907945 |
| HAUS6P3 | 2.5140267 | LRRC37A4P | -2.907945 |
| PSMC1P5 | 2.4794154 | FAM90A11P | -2.907945 |
| MTX1P1 | 2.4622888 | RPL21P44 | -2.907945 |
| RP11-297L17.6 | 2.4622888 | RP11-203L2.3 | -2.907945 |
| GRAMD4P8 | 2.4622888 | CD8BP | -2.907945 |
| ARMCX7P | 2.3784142 | AC073621.2 | -2.907945 |
| PTP4A2P2 | 2.3784142 | AC096579.13 | -2.907945 |
| UBA52P6 | 2.3784142 | OR8T1P | -2.907945 |
| SNRPEP2 | 2.3619853 | ENPP7P2 | -2.907945 |
| PTCHD3P1 | 2.3619853 | HYDIN2 | -2.907945 |
| IKBKGP1 | 2.3456699 | RP11-80I15.1 | -2.907945 |
| KRT8P10 | 2.3456699 | SLC25A14P1 | -2.907945 |
| RAET1K | 2.3456699 | RP11-386I23.1 | -2.907945 |
| KRT89P | 2.3456699 | RP11-266L9.3 | -2.907945 |
| RP11-216N14.7 | 2.3456699 | TPTE2P1 | -2.86791 |
| HMGB1P24 | 2.3456699 | SDHDP6 | -2.8481 |
| RAD17P1 | 2.3456699 | RPSAP41 | -2.8481 |
| RP11-561C5.4 | 2.3456699 | ULK4P1 | -2.828427 |
| KRT8P32 | 2.2973967 | TFP1 | -2.80889 |
| AC005795.1 | 2.2973967 | RP11-834C11.7 | -2.789487 |
| RPS15AP36 | 2.2815274 | RP11-798K23.5 | -2.770219 |
| RP11-481A20.10 | 2.2657678 | CYP4F29P | -2.770219 |
| ESPNP | 2.2657678 | DUX4L27 | -2.770219 |
| MGAT4EP | 2.2657678 | WHAMMP3 | -2.751084 |
| RP11-565J7.1 | 2.2657678 | RP11-530N7.2 | -2.751084 |
| NAT8B | 2.2345743 | RP11-137N23.1 | -2.732081 |
| CTD-2583A14.11 | 2.2191389 | CSPG4P12 | -2.732081 |
| COX20P1 | 2.2038102 | RNF138P1 | -2.732081 |
| SRSF9P1 | 2.2038102 | FAR2P2 | -2.732081 |
| PRDX1P1 | 2.2038102 | SNX18P12 | -2.694467 |
| RP11-465B22.3 | 2.1885874 | RBMS1P1 | -2.694467 |
| RP11-395L14.17 | 2.1584565 | RPS3AP34 | -2.675855 |
| TMEM191A | 2.1584565 | PGBD4P3 | -2.675855 |
| CASP16P | 2.1584565 | ANKRD62P1 | -2.675855 |
| PSME2P2 | 2.1435469 | HSPD1P11 | -2.675855 |
| RP11-564A8.4 | 2.1287404 | RP11-79D8.2 | -2.657372 |
| GMPSP1 | 2.1287404 | AC013271.3 | -2.657372 |
| RP11-815N9.2 | 2.1287404 | RP11-702H23.2 | -2.657372 |
| ZNF887P | 2.1140361 | RP11-511H9.3 | -2.657372 |
| CHMP4BP1 | 2.1140361 | TMED10P2 | -2.620787 |
| SUMO2P17 | 2.0994334 | CSPG4P11 | -2.620787 |
| SNX25P1 | 2.0994334 | PRSS44 | -2.620787 |
| CKS1BP3 | 2.0420243 | SIRPAP1 | -2.602684 |
| RP11-274E7.2 | 2.027919 | RP11-673E1.3 | -2.602684 |
| KRT8P31 | 2.0139111 | AC092641.2 | -2.602684 |
| KIF28P | 2.0139111 | SEPHS1P6 | -2.602684 |
| KRT18P17 | 2.0139111 | GBA3 | -2.602684 |
| RP11-537H15.4 | 2.0139111 | AC010980.2 | -2.602684 |
| PRSS29P | 2.0139111 | ACTG1P4 | -2.602684 |
| SLC9A3P3 | 2.0139111 | OR11H7 | -2.602684 |
| IFIT1P1 | 2.0139111 | SLED1 | -2.602684 |
| KRT8P37 | 2.0139111 | AC094019.4 | -2.602684 |
| CTD-2522E6.4 | 2.0139111 | MTND5P1 | -2.602684 |
| DHX9P1 | 2.0139111 | PSMC1P9 | -2.602684 |
| CCT5P1 | 2.0139111 | KRT17P1 | -2.602684 |
| IMPDH1P6 | 2.0139111 | DNM1P46 | -2.602684 |
| KRT8P49 | 2.0139111 | PKMP3 | -2.584706 |
| AC138035.3 | 2.0139111 | FABP5P7 | -2.584706 |
| KRT18P11 | 2.0139111 | RP11-632K20.7 | -2.566852 |
| RP11-763B22.3 | 2.0139111 | FGD5P1 | -2.531513 |
| HS6ST1P1 | 2.0139111 | RHOQP2 | -2.531513 |
| WBP11P1 | 2.0139111 | UNGP3 | -2.531513 |
| PYY2 | 2.0139111 | EIF3LP2 | -2.514027 |
| GAPDHP61 | 2.0139111 | AK3P3 | -2.514027 |
| EFTUD1P1 | 2.0139111 | EIF3EP1 | -2.514027 |
| RPL3P1 | 2.0139111 | RP11-32B5.1 | -2.514027 |
| KRT18P38 | 2.0139111 | PPP4R1L | -2.496661 |
| SERPINH1P1 | 2.0139111 | RP11-803B1.2 | -2.496661 |
| RP5-916O11.3 | 2.0139111 | FAM187B2P | -2.479415 |
| RP11-575G13.2 | 2.0139111 | RP11-146E13.5 | -2.479415 |
| RP11-21G15.1 | 2.0139111 | OR2A20P | -2.462289 |
| COL6A4P1 | 2.0139111 | IL6STP1 | -2.445281 |
| DNAJA1P3 | 2.0139111 | DNM1P47 | -2.445281 |
| MUC2 | 2.0139111 | BUD13P1 | -2.445281 |
| TLR12P | 2.0139111 | ATF4P1 | -2.445281 |
| RP11-415I12.2 | 2.0139111 | HSPA8P11 | -2.445281 |
| NANOGNBP3 | 2.0139111 | RP11-20I20.1 | -2.445281 |
| RP11-536C10.12 | 2.0139111 | CTC-559E9.9 | -2.445281 |
| ZNF725P | 2.0139111 | KARSP2 | -2.445281 |
| KRT8P36 | 2.0139111 | RP11-1081M5.3 | -2.445281 |
| RP5-854E16.2 | 2.0139111 | RP11-392E22.5 | -2.445281 |
| TNRC18P3 | 2.0139111 | EIF4A2P1 | -2.445281 |
| HSPB1P2 | 2.0139111 | SEPT10P1 | -2.445281 |
| AACSP1 | 2.0139111 | SNX18P3 | -2.445281 |
| KRT18P39 | 2.0139111 | AP000925.2 | -2.445281 |
| RP11-350E12.5 | 2.0139111 | ANKRD20A11P | -2.445281 |
| HLA-DPB2 | 2.0139111 | RP11-452D12.1 | -2.445281 |
| BPIFB9P | 2.0139111 | RP11-392A14.9 | -2.445281 |
| KRT8P50 | 2.0139111 | RP11-213G2.5 | -2.445281 |
| ABCB10P3 | 2.0139111 | ATF4P2 | -2.445281 |
| STIP1P3 | 2.0139111 | RP11-259P15.4 | -2.445281 |
| RP11-74M13.4 | 2.0139111 | AC005517.3 | -2.411616 |
| KRT18P25 | 2.0139111 | HERC2P4 | -2.411616 |
| KRT18P16 | 2.0139111 | ABCC6P1 | -2.411616 |
| TARDBPP2 | 2.0139111 | KRT87P | -2.378414 |
| KRT8P47 | 2.0139111 | SIGLEC17P | -2.378414 |
| RP11-586D19.1 | 2.0139111 | ZNF192P1 | -2.378414 |
| CICP9 | 2.0139111 | RP13-15M17.1 | -2.378414 |
| ENPP7P8 | 2.0139111 | CDCA4P1 | -2.378414 |
| TRIM80P | 2.0139111 | ALOX12P2 | -2.361985 |
| AP004290.1 | 2.0139111 | RPL23AP24 | -2.361985 |
| RP11-364B6.2 | 2.0139111 | RP11-578O24.2 | -2.34567 |
| LLNLR-304A6.2 | 2.0139111 | GVINP1 | -2.34567 |
| CXADRP3 | 2.0139111 | PCDHB19P | -2.34567 |
| FMO9P | 2.0139111 | CTD-3092A11.1 | -2.34567 |
| AC002056.3 | 2.0139111 | AK4P1 | -2.34567 |
| DCAF13P1 | 2.0139111 | bP-2171C21.4 | -2.34567 |
|  |  | RP11-392P7.1 | -2.329467 |
|  |  | CTD-2554C21.2 | -2.313376 |
|  |  | RP11-334A14.2 | -2.313376 |
|  |  | RP11-12A20.7 | -2.313376 |
|  |  | RPS2P32 | -2.313376 |
|  |  | AC016712.2 | -2.313376 |
|  |  | PCDHB18P | -2.297397 |
|  |  | RP11-475I24.9 | -2.297397 |
|  |  | CASP1P2 | -2.281527 |
|  |  | LINC01451 | -2.281527 |
|  |  | ULK4P3 | -2.281527 |
|  |  | RP11-597D13.7 | -2.281527 |
|  |  | HERC2P9 | -2.281527 |
|  |  | OR2A13P | -2.281527 |
|  |  | CLCN3P1 | -2.281527 |
|  |  | CXADRP1 | -2.281527 |
|  |  | CYP2F2P | -2.281527 |
|  |  | NPY6R | -2.281527 |
|  |  | HERC2P2 | -2.265768 |
|  |  | FAM27B | -2.265768 |
|  |  | DNAJC19P5 | -2.250117 |
|  |  | RPS4XP22 | -2.250117 |
|  |  | RP11-583F2.1 | -2.250117 |
|  |  | RP11-119F19.4 | -2.250117 |
|  |  | HMGN2P15 | -2.250117 |
|  |  | SNX18P13 | -2.234574 |
|  |  | RP11-989E6.13 | -2.234574 |
|  |  | TPT1P4 | -2.234574 |
|  |  | RHOQP3 | -2.234574 |
|  |  | PGAM1P5 | -2.234574 |
|  |  | RPS15AP12 | -2.234574 |
|  |  | SLC9A7P1 | -2.219139 |
|  |  | RBMXP4 | -2.219139 |
|  |  | RP11-537E18.1 | -2.219139 |
|  |  | SLC16A6P1 | -2.20381 |
|  |  | CEP164P1 | -2.20381 |
|  |  | AC019181.3 | -2.20381 |
|  |  | RP11-1180F24.1 | -2.20381 |
|  |  | RP11-157J24.1 | -2.20381 |
|  |  | YES1P1 | -2.20381 |
|  |  | GOLGA6L3 | -2.20381 |
|  |  | MTND6P4 | -2.20381 |
|  |  | MBL1P | -2.20381 |
|  |  | FRG1JP | -2.20381 |
|  |  | RNF126P1 | -2.20381 |
|  |  | RCN1P2 | -2.20381 |
|  |  | LDHAP2 | -2.20381 |
|  |  | GPAA1P2 | -2.188587 |
|  |  | PPIEL | -2.188587 |
|  |  | RP11-551L14.4 | -2.188587 |
|  |  | RP11-168A11.4 | -2.188587 |
|  |  | HIST2H2BB | -2.17347 |
|  |  | MST1L | -2.17347 |
|  |  | BEND3P3 | -2.17347 |
|  |  | CBX3P2 | -2.17347 |
|  |  | LPAL2 | -2.17347 |
|  |  | RP11-517P14.7 | -2.158456 |
|  |  | RPS9P1 | -2.158456 |
|  |  | RP11-712B9.5 | -2.158456 |
|  |  | TPTE2P6 | -2.158456 |
|  |  | IGLV1-41 | -2.158456 |
|  |  | ULK4P2 | -2.158456 |
|  |  | ZNF883 | -2.158456 |
|  |  | CH17-472G23.4 | -2.143547 |
|  |  | RP11-274B21.1 | -2.143547 |
|  |  | RP11-730A19.9 | -2.12874 |
|  |  | LINC00888 | -2.12874 |
|  |  | PGM5P2 | -2.114036 |
|  |  | RP11-1166P10.1 | -2.114036 |
|  |  | RPS15AP40 | -2.099433 |
|  |  | RPL18AP7 | -2.099433 |
|  |  | RPL21P120 | -2.099433 |
|  |  | RP11-420L9.2 | -2.099433 |
|  |  | MUC20P1 | -2.099433 |
|  |  | ZDHHC20P1 | -2.099433 |
|  |  | TEX21P | -2.099433 |
|  |  | RPL21P75 | -2.084932 |
|  |  | CH17-13I23.3 | -2.07053 |
|  |  | AC005154.8 | -2.07053 |
|  |  | DOC2GP | -2.07053 |
|  |  | HLA-J | -2.056228 |
|  |  | DEFB109P3 | -2.056228 |
|  |  | RP11-796G6.1 | -2.056228 |
|  |  | RP11-677M14.5 | -2.056228 |
|  |  | RPL5P29 | -2.042024 |
|  |  | RP11-266L9.6 | -2.042024 |
|  |  | HERC2P8 | -2.042024 |
|  |  | ZNF37BP | -2.042024 |
|  |  | RP11-12A20.4 | -2.042024 |
|  |  | RP11-603B24.1 | -2.027919 |
|  |  | CTB-33G10.1 | -2.027919 |
|  |  | RP11-110I1.5 | -2.027919 |
|  |  | VN1R108P | -2.027919 |
|  |  | CCDC144B | -2.027919 |
|  |  | OR2A9P | -2.027919 |
|  |  | UBE2FP3 | -2.027919 |
|  |  | POM121L9P | -2.027919 |
|  |  | RP1-199J3.5 | -2.013911 |
|  |  | PRDX3P1 | -2.013911 |
|  |  | RP11-632K20.2 | -2.013911 |
|  |  | SCML2P2 | -2.013911 |
|  |  | BX255923.2 | -2.013911 |
|  |  | LRRC37A17P | -2 |
|  |  | RP11-686D22.10 | -2 |
|  |  |  |  |

**Supplementary Table 2**. Dysregulated pseudogenes in BC using UALCAN database.

| GeneName | Expression | GeneName | Expression |
| --- | --- | --- | --- |
| CXCR2P1 | up | GGT3P | down |
| KLKP1 | up | HLA-J | down |
| RAET1K | up | CETN4P | down |
| PYY2 | up | ABCC6P1 | down |
| TMEM191A | up | ZNF204P | down |
| RPS26P11 | not-significant | PKD1L2 | down |
| RPLP0P2 | up | HERC2P4 | not-significant |
| MUC2 | up | POM121L9P | down |
| FMO9P | up | RPL21P44 | down |
| GUCY1B2 | up | MT1L | down |
| NAT8B | up | NPY6R | not-significant |
| WBP11P1 | up | FAM27B | not-significant |
| HNRNPA3P1 | up | LPAL2 | down |
| ESPNP | up | TPTE2P1 | down |
| HLA-DPB2 | up | RNF126P1 | down |
| CXADRP3 | up | RNF138P1 | down |
| ABCC13 | up | FER1L5 | down |
| TMED10P1 | up | HERC2P2 | down |
| OR7E91P | up | MBL1P | down |
| VN1R5 | up | PCDHB19P | down |
| CARD17 | up | RPS2P32 | down |
| HIST2H2BA | up | ZNF883 | down |
|  |  | CECR7 | down |
|  |  | RRN3P1 | down |
|  |  | PGM5P2 | down |
|  |  | CCDC144B | down |
|  |  | OR2A9P | down |
|  |  | PPIEL | down |
|  |  | GBA3 | not-significant |
|  |  | CASP12 | down |
|  |  | UBE2Q2P1 | down |
|  |  | SLED1 | down |
|  |  | PPP4R1L | down |
|  | | ALOX12P2 | not-significant |
|  |  |  |  |

**Supplementary Table 3.** Univariate analysis and multivariate analysis of the correlation of expression of HLA-DPB2 and HLA-DPB1 with overall survival among breast cancer patients.

| **Parameter** | **Univariate analysis** | | | **Multivariate analysis** | | | **Multivariate analysis** | | |
| --- | --- | --- | --- | --- | --- | --- | --- | --- | --- |
|  | **HR** | **95%CI** | **P-value** | **HR** | **95%CI** | **P-value** | **HR** | **95%CI** | **P-value** |
| Age | 1.04 | 1.02-1.05 | **1.13E-05** | 1.05 | 1.04-1.07 | **1.35E-09** | 1.05 | 1.04-1.07 | **2.17E-09** |
| Race | 1.25 | 0.87-1.79 | 0.221 | 1.79 | 1.22-2.62 | **0.003** | 1.52 | 1.04-2.22 | **0.029** |
| Histological type | 1.07 | 0.96-1.20 | 0.221 |  |  |  |  |  |  |
| T classificant | 1.48 | 1.16-1.89 | **0.002** | 0.86 | 0.60-1.23 | 0.405 | 0.84 | 0.58-1.21 | 0.352 |
| N classificant | 1.66 | 1.35-2.04 | **1.69E-06** | 0.90 | 0.62-1.29 | 0.550 | 0.94 | 0.65-1.34 | 0.724 |
| M classificant | 7.64 | 3.95-14.75 | **1.43E-09** | 0.74 | 0.28-1.99 | 0.551 | 0.58 | 0.21-1.61 | 0.297 |
| Stage | 2.12 | 1.63-2.77 | **2.75E-08** | 1.87 | 0.98-3.58 | 0.058 | 1.76 | 0.92-3.34 | 0.086 |
| Tumor status | 11.42 | 7.60-17.16 | **1.11E-31** | 12.28 | 7.91-19.08 | **6.47E-29** | 12.47 | 8.01-19.40 | **4.89E-29** |
| HLA-DPB2 | 0.74 | 0.60-0.92 | **0.006** | 0.66 | 0.51-0.86 | **0.002** |  |  |  |
| HLA-DPB1 | 0.995 | 0.9918-0.9988 | **0.009** |  | | | 0.996 | 0.9928-0.9995 | **0.025** |

Bold values indicate P ＜0.05，HR, hazard ratio; CI, confidence interval.

**Supplementary Table 4.** Significant changes of has-miR-370-3p expression between breast cancer and normal tissues (dbDEMC 2.0). adj: adjust.

| Experiment ID | Cancer Type | Cancer Subtype | Design | LogFC | AveExpr | T-value | P-value | adj P-value | Status |
| --- | --- | --- | --- | --- | --- | --- | --- | --- | --- |
| GSE4589 | breast cancer |  | cancer vs normal | 2.32 | 2.32 | 7.09 | 7.56E-08 | 2.27E-07 | UP |
| GSE40525 | breast cancer |  | cancer vs normal | -0.88 | 7.00 | -3.17 | 1.94E-03 | 6.30E-03 | DOWN |
| GSE40525 | breast cancer | ER negative | cancer vs normal | -1.49 | 7.00 | -3.32 | 1.22E-03 | 5.61E-03 | DOWN |
| GSE40525 | breast cancer | PR negative | cancer vs normal | -1.10 | 7.00 | -3.09 | 2.47E-03 | 9.30E-03 | DOWN |
| GSE45666 | breast cancer |  | cancer vs normal | -1.28 | 3.06 | -3.58 | 4.90E-04 | 1.08E-03 | DOWN |
| GSE45666 | breast cancer | ER positive | cancer vs normal | -1.47 | 3.06 | -3.85 | 1.93E-04 | 4.50E-04 | DOWN |
| GSE45666 | breast cancer | ER negative | cancer vs normal | -1.10 | 3.06 | -2.95 | 3.80E-03 | 7.65E-03 | DOWN |
| GSE45666 | breast cancer | PR positive | cancer vs normal | -1.59 | 3.06 | -4.01 | 1.07E-04 | 2.67E-04 | DOWN |
| GSE45666 | breast cancer | PR negative | cancer vs normal | -1.10 | 3.06 | -3.03 | 3.02E-03 | 6.17E-03 | DOWN |
| GSE45666 | breast cancer | HER2+ positive | cancer vs normal | -1.60 | 3.06 | -4.05 | 9.40E-05 | 2.40E-04 | DOWN |
| GSE45666 | breast cancer | HER3+ negative | cancer vs normal | -1.09 | 3.06 | -3.00 | 3.31E-03 | 6.68E-03 | DOWN |
| GSE38167 | breast cancer |  | cancer vs normal | -0.48 | 2.82 | -2.97 | 4.12E-03 | 2.14E-02 | DOWN |
| GSE61438 | breast cancer |  | cancer vs normal | -2.01 | 9.49 | -4.05 | 1.29E-04 | 1.14E-03 | DOWN |
| TCGA_BRCA | breast cancer | breast invasive carcinoma | cancer vs normal | -0.27 | 1.44 | -6.62 | 5.54E-11 | 1.44E-10 | DOWN |
|  |  |  |  |  |  |  |  |  |  |

**Supplementary Table 5.** The top 100 correlated genes of HLA-DPB2 and HLA-DPB1 obtained from UALCAN database. CC, correlation coefficient.

| HLA-DPB2 | Expression | Pearson-CC | HLA-DPB1 | Expression | Pearson-CC |
| --- | --- | --- | --- | --- | --- |
| HLA-DRA | up | 0.58 | HLA-DRA | up | 0.88 |
| HLA-DMB | up | 0.57 | HLA-DPA1 | up | 0.88 |
| HLA-DMA | not-significant | 0.56 | HLA-DMA | not-significant | 0.87 |
| HLA-DPB1 | up | 0.56 | HLA-DMB | up | 0.85 |
| CD74 | up | 0.55 | CD74 | up | 0.85 |
| CIITA | down | 0.53 | MYO1F | up | 0.84 |
| HLA-DOA | not-significant | 0.52 | LST1 | up | 0.83 |
| AMICA1 | not-significant | 0.52 | HLA-DRB1 | up | 0.82 |
| HLA-DPA1 | up | 0.51 | SELPLG | up | 0.82 |
| CST7 | up | 0.5 | FERMT3 | up | 0.82 |
| SPN | up | 0.5 | HCLS1 | up | 0.82 |
| ARHGAP30 | up | 0.49 | FMNL1 | up | 0.8 |
| LCK | up | 0.49 | TNFRSF1B | down | 0.8 |
| PTPN7 | up | 0.49 | AMICA1 | not-significant | 0.8 |
| C17orf87 | up | 0.49 | ABI3 | up | 0.8 |
| CD53 | up | 0.49 | WAS | up | 0.8 |
| IL18BP | up | 0.49 | TNFAIP8L2 | up | 0.78 |
| IL12RB1 | up | 0.49 | CYTH4 | not-significant | 0.78 |
| WAS | up | 0.49 | ARHGAP30 | up | 0.78 |
| CD6 | up | 0.49 | FAM78A | up | 0.78 |
| CRTAM | up | 0.49 | DOK2 | down | 0.78 |
| HCLS1 | up | 0.49 | HLA-DOA | not-significant | 0.77 |
| IL2RG | up | 0.48 | GMFG | down | 0.77 |
| CD247 | down | 0.48 | NCF4 | not-significant | 0.77 |
| HLA-DOB | down | 0.48 | CD4 | up | 0.77 |
| ARHGAP9 | up | 0.48 | AIF1 | not-significant | 0.77 |
| SIRPG | up | 0.48 | IL10RA | not-significant | 0.77 |
| NCF1 | up | 0.48 | HCST | not-significant | 0.77 |
| CTSW | down | 0.48 | RASAL3 | up | 0.77 |
| HLA-DRB1 | up | 0.48 | HLA-E | down | 0.77 |
| HCST | not-significant | 0.48 | GNGT2 | up | 0.76 |
| CD3D | up | 0.48 | SPI1 | up | 0.76 |
| FERMT3 | up | 0.48 | CIITA | down | 0.76 |
| HLA-DQA1 | up | 0.48 | MFNG | down | 0.76 |
| BIN2 | up | 0.48 | TMEM149 | up | 0.76 |
| HLA-E | down | 0.48 | SASH3 | up | 0.76 |
| SASH3 | up | 0.48 | DOCK2 | up | 0.75 |
| TNFRSF1B | down | 0.47 | ARHGAP9 | up | 0.75 |
| CD96 | down | 0.47 | IL12RB1 | up | 0.75 |
| MYO1F | up | 0.47 | GIMAP1 | down | 0.75 |
| TNFAIP8L2 | up | 0.47 | CD6 | up | 0.75 |
| LCP2 | up | 0.47 | SPN | up | 0.75 |
| PARVG | up | 0.47 | HLA-DRB6 | up | 0.75 |
| UBASH3A | up | 0.47 | CORO1A | up | 0.74 |
| NKG7 | down | 0.47 | PARVG | up | 0.74 |
| KLRK1 | not-significant | 0.47 | PTPN7 | up | 0.74 |
| GZMA | up | 0.47 | CXCR3 | up | 0.74 |
| CD2 | up | 0.47 | SNAI3 | up | 0.74 |
| CXCR3 | up | 0.47 | EBI3 | up | 0.74 |
| CD8A | down | 0.47 | GAB3 | down | 0.74 |
| CXCR6 | up | 0.47 | HLA-DQB1 | up | 0.74 |
| TIGIT | up | 0.47 | CST7 | up | 0.73 |
| TMEM149 | up | 0.47 | ITGB2 | up | 0.73 |
| CD48 | up | 0.47 | CD247 | down | 0.73 |
| CD3E | up | 0.47 | PLCB2 | up | 0.73 |
| SNX20 | up | 0.47 | CD53 | up | 0.73 |
| XCL2 | down | 0.47 | NCF1 | up | 0.73 |
| FMNL1 | up | 0.46 | CD2 | up | 0.73 |
| IGSF6 | up | 0.46 | AOAH | down | 0.73 |
| ITGB7 | up | 0.46 | GPSM3 | up | 0.72 |
| CORO1A | up | 0.46 | LCP2 | up | 0.72 |
| GNGT2 | up | 0.46 | NAPSB | up | 0.72 |
| IL21R | up | 0.46 | CARD11 | up | 0.72 |
| SLA2 | up | 0.46 | LCK | up | 0.72 |
| DOCK2 | up | 0.46 | PIK3R5 | not-significant | 0.72 |
| CD5 | up | 0.46 | FGD2 | not-significant | 0.72 |
| CD4 | up | 0.46 | CTSW | down | 0.72 |
| FAM78A | up | 0.46 | GIMAP5 | down | 0.72 |
| FGD2 | not-significant | 0.46 | PSTPIP1 | up | 0.72 |
| INPP5D | down | 0.46 | CD3D | up | 0.72 |
| JAK3 | up | 0.46 | CRTAM | up | 0.72 |
| IL10RA | not-significant | 0.46 | APBB1IP | down | 0.72 |
| MAP4K1 | up | 0.46 | LAT | up | 0.71 |
| LST1 | up | 0.46 | IL21R | up | 0.71 |
| PLEK | up | 0.46 | CD5 | up | 0.71 |
| CCR5 | up | 0.46 | MS4A6A | down | 0.71 |
| ARHGAP25 | down | 0.46 | UBASH3A | up | 0.71 |
| AOAH | up | 0.46 | S1PR4 | up | 0.71 |
| BTK | up | 0.45 | HLA-DQA1 | up | 0.71 |
| KLHL6 | up | 0.45 | LSP1 | not-significant | 0.71 |
| LAT | up | 0.45 | C10orf54 | down | 0.71 |
| GRAP2 | not-significant | 0.45 | AKNA | down | 0.71 |
| SLAMF1 | down | 0.45 | SNX20 | up | 0.71 |
| LCP1 | up | 0.45 | GRAP2 | not-significant | 0.7 |
| LTA | up | 0.45 | ACAP1 | up | 0.7 |
| SLAMF7 | up | 0.45 | GZMA | up | 0.7 |
| ACAP1 | up | 0.45 | IL18BP | down | 0.7 |
| EVI2B | up | 0.45 | C1QA | not-significant | 0.7 |
| DOK2 | down | 0.45 | CCR5 | up | 0.7 |
| SIT1 | up | 0.45 | PLEKHO2 | down | 0.7 |
| FAM26F | up | 0.45 | NCKAP1L | up | 0.7 |
| SP140 | up | 0.45 | VAV1 | up | 0.7 |
| LY9 | down | 0.45 | BTK | up | 0.69 |
| SLAMF8 | up | 0.45 | LAIR1 | down | 0.69 |
| PRF1 | not-significant | 0.45 | SLA2 | up | 0.69 |
| SELPLG | up | 0.45 | NKG7 | down | 0.69 |
| HLA-DQA2 | up | 0.45 | LAT2 | up | 0.69 |
| IRF8 | not-significant | 0.45 | SIT1 | up | 0.69 |
| CD27 | up | 0.45 | MYO1G | up | 0.69 |
|  |  |  |  |  |  |
